# An Ultrasoft and Flexible PDMS-Based Balloon-Type Implantable Device for Controlled Drug Delivery

**DOI:** 10.34133/bmr.0012

**Published:** 2024-03-28

**Authors:** Tausif Muhammad, Byungwook Park, Aseer Intisar, Minseok S. Kim, Jin-Kyu Park, Sohee Kim

**Affiliations:** ^1^ Department of Robotics and Mechatronics Engineering, Daegu Gyeongbuk Institute of Science and Technology, Daegu 42988, Republic of Korea.; ^2^ Department of New Biology, Daegu Gyeongbuk Institute of Science and Technology, Daegu 42988, Republic of Korea.; ^3^ Department of Veterinary Pathology, College of Veterinary Medicine, Kyungpook National University, Daegu 41566, Republic of Korea.

## Abstract

Non-biodegradable implants have undergone extensive investigation as drug delivery devices to enable advanced healthcare toward personalized medicine. However, fibroblast encapsulation is one of the major challenges in all non-biodegradable implants, besides other challenges such as high initial burst, risk of membrane rupture, high onset time, non-conformal contact with tissues, and tissue damage. To tackle such challenges, we propose a novel ultrasoft and flexible balloon-type drug delivery device for unidirectional and long-term controlled release. The ultrasoft balloon-type device (USBD) was fabricated by using selective bonding between 2 polydimethylsiloxane (PDMS) membranes and injecting a fluid into the non-bonded area between them. The balloon acted as a reservoir containing a liquid drug, and at the same time, the membrane of the balloon itself acted as the pathway for release based on diffusion. The release was modulated by tuning the thickness and composition of the PDMS membrane. Regardless of the thickness and composition, all devices exhibited zero-order release behavior. The longest zero-order release and nearly zero-order release were achieved for 30 days and 58 days at a release rate of 1.16 μg/day and 1.68 μg/day, respectively. In vivo evaluation was performed for 35 days in living rats, where the USBD maintained zero-order and nearly zero-order release for 28 days and 35 days, respectively. Thanks to the employment of ultrasoft and flexible membranes and device design, the USBD could achieve minimal tissue damage and foreign body responses. It is expected that the proposed device may provide a novel approach for long-term drug delivery with new therapeutic modalities.

## Introduction

Oral, transdermal, and intravenous administrations of drugs are the most popular drug delivery methods. However, first-pass metabolism, degradation in the stomach, poor bioavailability, and patient compliance are inherent disadvantages [1,2]. With advancements in micro- and nano-technology, drug delivery implants have emerged as a potential alternative to the conventional administration of drugs for a sustained and controlled release of drugs to make the existing therapies more efficient with minimal side effects [3–5].

Several of these systems have been fabricated with biocompatible polymer compositions, where drug diffusion and polymer degradation act primarily to modulate drug release [6]. Among these implantable systems, non-biodegradable implants (i.e., reservoir-type, matrix-type, and active implants) are well established [7]. However, there are limitations, including membrane rupture [8–10], a leading cause of drug dumping, which may result in unexpected toxic side effects. Also, a high onset time [11,12] causes an excessive delay in drug release. Such implants utilize drugs in powder form, where water infiltrates via a channel into the drug reservoir to dissolve the drug, and then the drug molecules could diffuse out for sustained drug release. In addition, these implantable devices are often associated with high initial burst release [13,14], affecting long-term efficacy and zero-order drug release [15,16], which limits their use for long-term drug delivery. Most reservoir-based implantable systems are manufactured via molding of various soft polymers integrated with a permeable membrane, making them relatively rigid and thick [11,17,18]. Consequently, the mechanical mismatch between biological tissues and implantable devices could cause tissue damage, resulting in severe foreign body responses [19]. Also, non-conformal contact with biological tissue limits target drug delivery and may degrade the release kinetics of the device at the implantation site. All these limitations including high initial burst, risk of membrane rupture, high onset time, non-conformal contact with biological tissues, tissue damage, and foreign body responses still need to be minimized by introducing novel devices in the field of localized drug delivery.

**Fig. 1.**
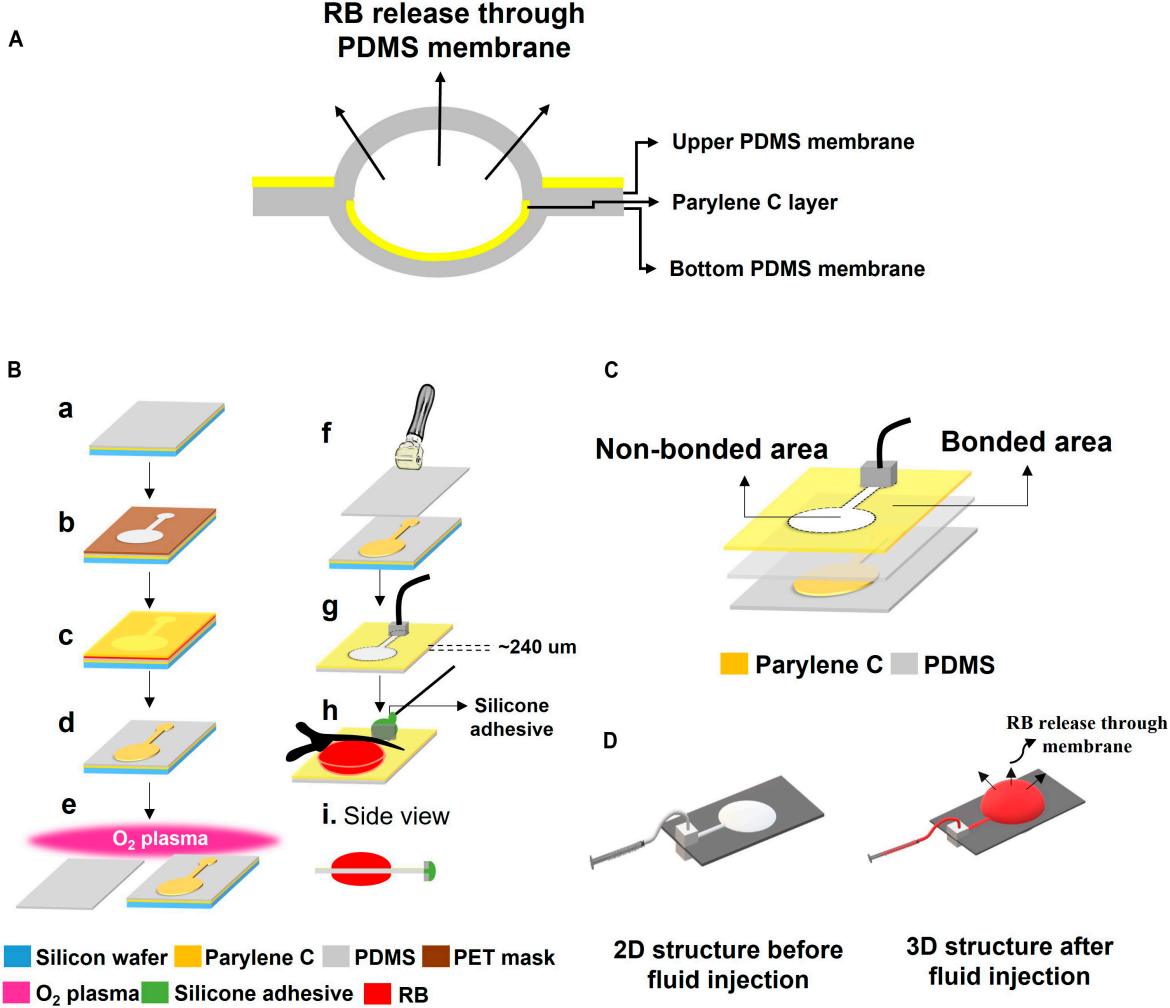
Schematic illustration of the USBD fabrication: (A) Release mechanism and cross-sectional view of the USBD, (B) fabrication processes of the USBD [(a) parylene C deposition and bottom PDMS layer spin coating; (b to d) patterned parylene C deposition for selective bonding; (e) surface activation of top and bottom PDMS layers using oxygen plasma; (f) selective bonding of top and bottom PDMS layers; (g) insulation of bonded area, PDMS block attachment, and silicone tube insertion for fluid injection; (h) closing inlet channel with silicone adhesive; (i) side view of the complete USBD after RB injection], (C) schematics of the layers that compose the final device, and (D) generation of a 3D structured reservoir after fluid injection.

Here, we propose an ultrasoft balloon-type device (USBD) specifically aiming to minimize foreign responses, which is one of the most prominent problems in non-biodegradable implants along with solving the aforementioned problems in previously developed devices. The USBD is fabricated using polydimethylsiloxane (PDMS) and poly(p-xylylene) (parylene C) by selective bonding through plasma treatment on the selected areas [20]. These materials have been widely used in implantable devices due to their high biocompatibility, classified as United States Pharmacopeia class VI, high flexibility, low stiffness, long-term biostability under physiological temperature and pH fluctuations, and mechanical properties being close to biological tissues [21–29]. The device consists of a balloon reservoir made of 2 PDMS membranes selectively bonded together, and an inlet channel through which drugs can be injected into the reservoir. By injecting the drug in solution form into the reservoir, the USBD in a 2-dimensional (2D) plane turns into a 3-dimensional (3D) balloon-type reservoir; herein, the upper PDMS membrane functions as a drug diffusion membrane, and the lower PDMS membrane patterned with parylene C prevents drug diffusion, ensuring unidirectional controlled release (Fig. 1A). Rhodamine B (RB), a fluorescence material, is employed as a model release agent to investigate the viability of the USBD in both in vitro drug release study and in vivo pharmacokinetic experiments. The biocompatibility of the USBD is investigated using in vitro cell viability test as well as histopathological analysis after in vivo implantation for 35 days.

## Materials and Methods

### Materials

PDMS (Sylgard-184) was purchased from Dow Corning (Midland, MI, USA). Parylene C dimer was obtained from Nuri-Tech (Incheon, Korea). Silicone adhesive (Kwik-Sil & Kwik-Cast) were purchased from World Precision Instrument (Sarasota, FL, USA). Silicone tubes were purchased from Saint-Gobain (Solon, OH, USA). Phosphate buffered saline (PBS: pH 7.4), 10% fetal bovine serum (FBS), and Dulbecco’s Modified Eagle’s Medium (DMEM) were purchased from Welgene (Gyeongsan, Korea). RB (99.0% purity; molecular weight [MW]: 479.01 g/mol; water solubility: 1 mg/ml), antibiotic–antimycotic, ethanol, and xylene were purchased from Sigma (St. Louis, MO, USA). Isoflurane was obtained from Hana Pharm (Seoul, Korea). Meloxicam was purchased from Fuoviders (Ansan, Korea). Enrofloxacine (Enromax) was obtained from Woogene B&G (Seoul, Korea). Paraformaldehyde (4%) was purchased from Han Lab (Cheongju, Korea). Hematoxylin and eosin (H&E) solution was purchased from Abcam (Cambridge, UK).

### USBD fabrication

#### Fabrication of selectively patterned 2D structure

The fabrication of the USBD is illustrated in Fig. 1B (a to i). The USBD is composed of 4 layers: the bottom PDMS layer, the intermediate parylene C pattern, the top PDMS layer, and the final parylene C pattern as depicted in Fig. 1B. To fabricate the USBD, parylene C was first deposited on a silicon wafer as a sacrificial layer using a parylene coater (NRPC-500, Nuri Tech Corp., Incheon, Korea). After that, PDMS was mixed at a weight ratio of 1:10 (curing agent:monomer) and degassed in a desiccator followed by spin coating the bottom PDMS layer at 300 rpm for 60 s and curing in a dry oven at 120 °C for 1 h (Fig. 1B, a). For selective bonding [20], the intermediate parylene C layer was deposited after placing a reverse polyethylene terephthalate (PET) mask patterned with a CO_2_ laser (VLS3.75, Universal Laser Systems, Scottsdale, AZ, USA) on the first PDMS layer (Fig. 1B, b). After parylene C deposition, the PET mask was detached from the bottom PDMS layer, leaving behind the patterned parylene C (Fig. 1B, c and d). The top PDMS membrane was fabricated separately, which acts as a semi-permeable membrane for the controlled release of RB. For selective bonding of the top and bottom layers of PDMS, both surfaces were treated with O_2_ plasma in a plasma system (CUTE, Femto Science, Hwaseong, Korea) using an oxygen flow rate of 40 standard cubic centimeters per minute (sccm) and 100 W power for 1 min (Fig. 1B, e). Then, the 2 layers were bonded together manually using rubber roller to avoid trapped bubbles (Fig. 1B, f). To increase the bonding strength, the device was cured in an oven for 10 s at 80 °C followed by the deposition of the final parylene C layer on the bonded area to prevent the back diffusion of RB into the PDMS layers (Fig. 1B, g). The selectively bonded 2D structure was peeled off from the substrate for further processing. The schematics of the layers that compose the final device are shown in Fig. 1C.

#### Generation of 3D reservoir by fluid injection

After the fabrication of a selectively bonded structure in a 2D plane, PDMS blocks with a size of 3 mm × 3 mm × 3 mm were covalently bonded to both the top and bottom PDMS layers by oxygen plasma treatment at an oxygen flow rate of 40 sccm and 100 W power for 1 min (Fig. 1B, g). An inlet hole was punched in the upper PDMS block using a biopsy punch (Rapid-Core 1.2 mm; WPI, Sarasota, FL, USA). A silicone tube was inserted into the punched hole, and a syringe was connected to the other end of the silicone tube for RB solution injection. After that, RB solution was injected into the non-bonded area of the USBD using a syringe pump (Fusion 100, Chemyx Inc., Stafford, TX, USA) at a rate of 15 μl/min. As a result, fluid injection into the non-bonded area turned it into a 3D balloon-type reservoir, as shown in Fig. 1D. After injecting the target amount of RB, the inlet channel was closed using Kwik-Cast silicone adhesive (Fig. 1B, h and i).

#### USBDs with different thicknesses and compositions

The USBDs were prepared with various PDMS membranes, where the membrane thickness and mixing ratio were varied to achieve various release kinetic profiles. Thus, the USBDs with membrane thicknesses of 255, 178, 140, 115, 96, 81, and 73 μm were designated as USBD255, USBD178, USBD140, USBD115, USBD96, USBD81, and USBD73, respectively. In addition, another set of release kinetic profiles was investigated where mixing ratios of 1:5, 1:10, 1:15, 1:20, and 1:25 were used to prepare USBD5, USBD10, USBD15, USBD20, and USBD25, respectively.

### USBD characterization

The bonded and non-bonded areas of the fabricated USBD were examined by field-emission scanning electron microscopy (FE-SEM SU8020, Hitachi High-Technologies, Tokyo, Japan). Scanning electron microscopy–energy dispersive x-ray spectroscopy (SEM-EDS) analysis was also conducted for cross-sectional mapping of the USBD. For this, the USBD was broken using liquid nitrogen to reveal a cross-section of the inlet channel. Then, it was placed on a sample mount and sputter-coated with 1 to 3 nm platinum for 10 min. To evaluate the initial drug loading capacity, each USBD with various membrane thicknesses and compositions was filled with RB solution and immediately immersed in 20 ml of PBS to fully dissolve the RB. The aliquot was collected and measured at 551 nm using an ultraviolet–visible spectrophotometer (UV-Vis-NIR; Cary 5000, Agilent Technologies, Santa Clara, CA, USA). To assess the structural integrity of the USBD, each USBD was filled with RB solution to the maximum extent just below the burst point and immersed in PBS to examine leakage and balloon burst. Similarly, the maximum loading capacity for each USBD was optimized to the point where no structural failure was observed and all USBDs survived for up to 5 months or longer.

### In vitro release study

For the in vitro study, RB solution (0.5 %w/v, solvent: Ultra-deionized water [DI] water), a fluorescence agent, was used as a model drug to easily visualize the release kinetics. Each USBD was filled with RB and fully immersed in 15 ml of pH 7.4 PBS acting as a receiving phase at 37 °C. To estimate the amount of RB diffused out of the USBD (*n* = 4 for each release kinetic profile) into the PBS, aliquots were collected at scheduled intervals over 5 months and measured spectrophotometrically at 551 nm using a UV-Vis spectrophotometer. After collecting the aliquots, previously used PBS was replenished with fresh PBS to avoid RB saturation in the receiving phase. Concentrations of the collected aliquots were calculated by comparing the absorbance peak to the standard curves made with known concentrations of standard solutions.

### Cytotoxicity test

To investigate the cell compatibility with the USBD and its constituent materials, a direct in vitro cytotoxicity test [30] was performed on empty USBDs with and without silicone adhesive. Cell viability was evaluated by a cell counting kit (CCK-8; Dojindo lab, Kumamoto, Japan) using C_2_C_12_ mouse myoblast cells (ATCC, Manassas, VA, USA). Briefly, each USBD was sterilized with ethylene oxide followed by UV sterilization for 30 min. It was inserted in individual wells of a 12-well plate (Eppendorf, Hamburg, Germany) in a way where the releasing PDMS membrane was facing upward. Then, C_2_C_12_ cells were seeded on each USBD surface at a density of 20,000 cells per well. Cell proliferation on a bare polystyrene surface of a 12-well plate without the USBD was set as the control. Cell culture was carried out for 1 week using DMEM with 10% FBS and 1% antibiotic–antimycotic in an incubator with 5% CO_2_ at 37 °C. Cell viability was measured on culture days 1, 3, 5, and 7 using a CCK-8 kit, in which 10 μl of WST-8 (CCK-8 kit) solution in each well (100 μl medium) was added and incubated for 2 h at 37 °C according to the manufacturer's instructions. After incubation, 100 μl of the mixture from each well was separated and placed inside individual wells of a 96-well plate (Corning Inc., Corning, NY, USA). Subsequently, absorbance was measured at 450 nm for each well by a microplate reader (VersaMax, Molecular Devices, San Jose, CA, USA).

### In vivo evaluation

#### Animal maintenance

In this study, male Sprague–Dawley rats aged 5 to 7 weeks weighing 180 to 250 g and male nude BALB/c mice aged 10 to 15 weeks weighing 20 to 27 g were used and maintained under specific-pathogen-free (SPF) conditions in the Laboratory Animal Resource Center (LARC), following the experimental protocols approved by the Institutional Animal Care and Use Committee (Approval No. DGIST-IACUC 23012602-0004) at Daegu Gyeongbuk Institute of Science and Technology (DGIST).

#### USBD implantation

For implantation of the USBD, animals were anesthetized using a respiratory anesthetic system (MatrxTM VIP 3000, Midmark, OH, USA). Anesthesia was induced in a chamber with 5.0% isoflurane for 3 min and maintained with inhalation of 3% isoflurane through a mask during the surgical procedure. The hair on the dorsal region was shaved and disinfected with betadine. After that, a skin incision of 6 to 7 mm was made, and the USBD sterilized with hydrogen peroxide followed by ethylene oxide was implanted in the subcutaneous pocket (Fig. S1). During implantation, the USBD was carefully oriented such that the PDMS membrane releasing RB was faced toward the subcutaneous muscles. The incision was sutured with a nylon 4-0 thread (NB434, Ailee Sutures, Busan, Korea), and the surgical site was disinfected with betadine. After surgery, anti-inflammatory Meloxicam (0.01 ml/100 g) and antibiotic Enrofloxacin (0.025 ml/100 g) were injected into the muscle.

#### In vivo release study

To perform in vivo pharmacokinetic evaluation, the implanted USBDs were extracted from rats at scheduled time intervals (*n* = 3) [31,32] and immediately washed with acetone, isopropanol, ethanol, and ultra-DI water to remove residues from the device surface. After that, each USBD was cut open in 20 ml of PBS and left overnight in a rotary mixture (Programmable Digital Rotator RT-10, Daihan Scientific Co., Wonju, Korea) to completely dissolve the residual RB in PBS. Aliquots of 5 ml were collected from the solution and measured spectrophotometrically at 551 nm using a UV-Vis spectrophotometer as discussed previously. As a control, 5 USBDs were filled with RB mimicking the in vivo setup, after which the RB in the balloon reservoir was diluted with 20 ml of PBS, and the average initial amount was calculated for each implanted USBD. The amount of released RB was calculated by subtracting the residual amount from the initial amount.

#### Histopathological evaluation

The implanted rats (*n* = 4) were euthanized by CO_2_ inhalation, and the tissue in the dorsal region around the USBD was harvested after 35 days of implantation. The retrieved USBDs surrounded by tissue were fixed in 4% paraformaldehyde in a conical tube for 24 h at 4 °C. Fixed tissues were embedded with paraffin wax where care was taken to orient the sample such that histological sections of the device cross-sections could be obtained to reveal the thickness of the fibrotic capsule. After that, the paraffinized samples were sliced into 5-μm-thick slices and mounted on glass slides. The slides were deparaffinized with xylene, rehydrated with ethanol in descending order, and washed with distilled water. For staining, slides were processed with hematoxylin solution for 5 min and rinsed with distilled water followed by the bluing agent for 10 s and rinsing with distilled water and 100% ethyl alcohol. Afterwards, the slices were treated with eosin Y solution for 3 min and rinsed with ethyl alcohol in ascending order (70%, 80%, 90%, and 100%). To investigate the degree of inflammation and capsule thickness, slices with USBD cross-sections from 3 different regions were obtained as follows: inlet channel, reservoir, and bonded membranes to completely assess the minimum, maximum, and average capsule thicknesses. Three images of each slice were taken at 4× magnification to cover the entire cross-section of the USBD. Thus, a total of 36 images were obtained per animal group (*n* = 4). The stained slices were assessed by a professional pathologist using an upright motorized microscope (Eclipse Ni-E, Nikon, Tokyo, Japan) at 200× magnification.

#### Masson’s trichrome staining

Deparaffinized and rehydrated slices were stained in Weigert's iron hematoxylin working solution for 10 min followed by washing in distilled water and staining in Biebrich scarlet-acid fuchsin solution for 15 min. After that, the slices were differentiated in phosphomolybdic–phosphotungstic acid solution for 15 min and then transferred directly to aniline blue solution for 5 to 10 min. The slices were briefly rinsed in distilled water, differentiated in 1% acetic acid for 1 min and again washed in distilled water. The stained slices were then dehydrated gradually through 80% ethyl alcohol and cleared in toluene. The degree of inflammation and capsule thickness based on collagen density were assessed as discussed previously. All slices were assessed by a professional pathologist using an upright motorized microscope (Eclipse Ni-E, Nikon, Japan) at 200× magnification.

#### IVIS imaging

To visualize the in vivo RB release from the USBD over time, an in vivo imaging system (IVIS; IVIS® Spectrum Series, ParkinElmer, Waltham, MA, USA) was used. In total, 4 nude mice were implanted with USBDs: 3 mice were implanted with RB-filled devices and one was implanted with empty USBD acting as a control. IVIS imaging was performed for 55 days at predetermined time intervals of 7 days. The images were obtained with excitation and emission wavelengths of 570 and 680 nm, respectively.

#### Statistical analysis

All experimental data were expressed as the mean ± standard deviation. Statistical significance in the data were calculated by one-way analysis of variance using Tukey’s multiple comparisons test using Origin 2017 Software (Origin Lab Corp., Northampton, MA, USA). Differences were considered statistically significant if *P* < 0.05 (*).

## Results

### Fabrication and characterization of USBD

**Fig. 2.**
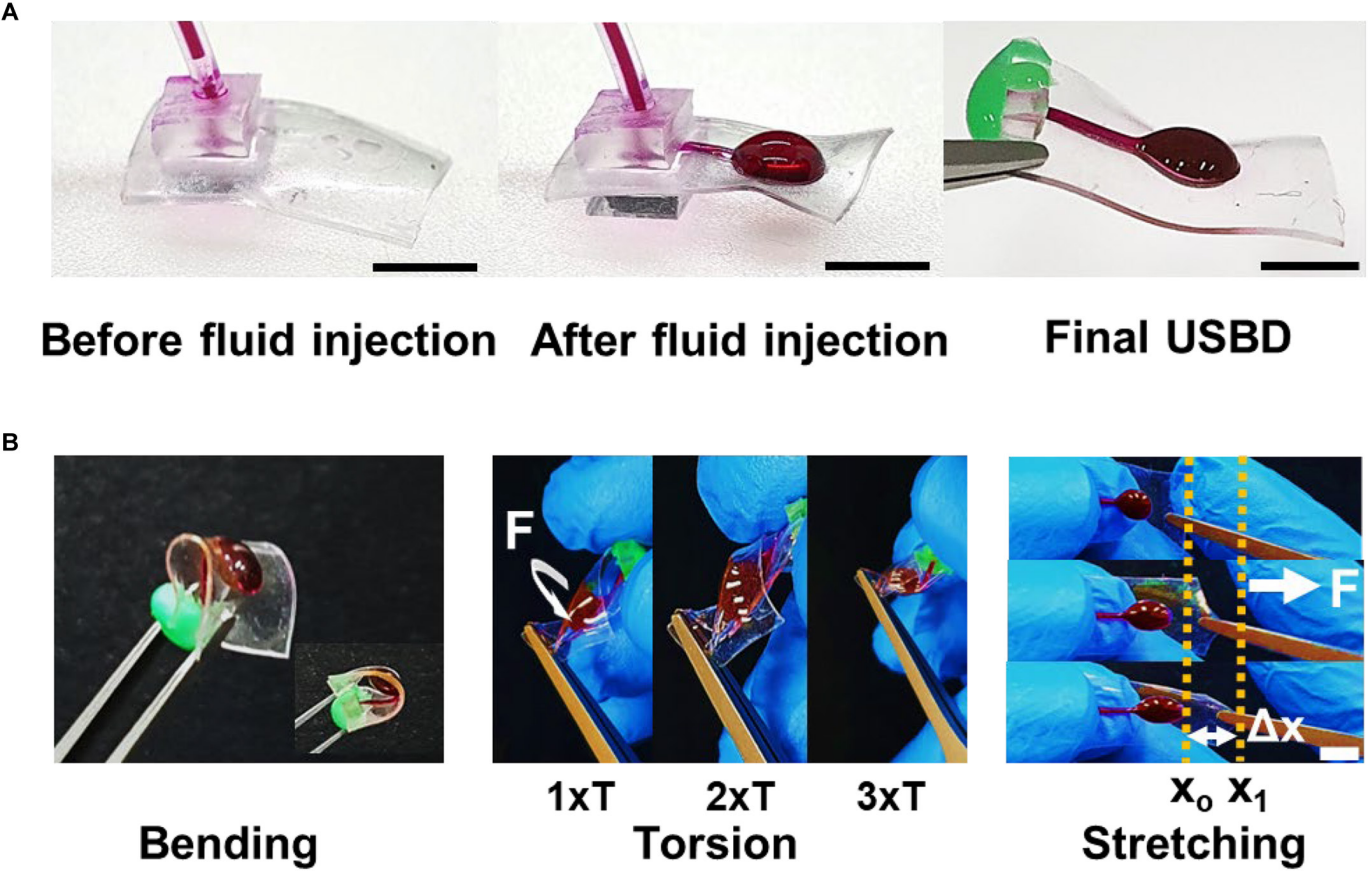
Optical images of the USBD and its softness and flexibility: (A) USBD before and after the injection of RB solution, and (B) images to show its ultrasoft and flexible properties, by applying bending, torsional, and stretching forces. 1xT, 2xT, and 3xT represent the number of twists, and Δ*x* represents the change in length when the USBD was stretched longitudinally, which was 6 mm (35% tensile strain). Scale bars are 5 mm.

**Fig. 3.**
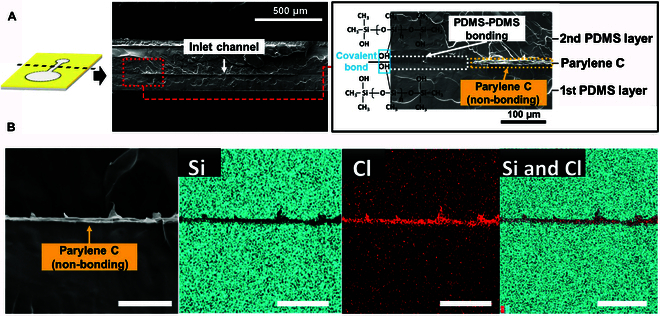
Cross-sectional analysis of the USBD using SEM-EDS: (A) SEM images of the cross-section of the USBD, showing the difference between the bonded and non-bonded regions. (B) SEM-EDS analysis of the cross-section of the USBD, showing the atomic distribution of the non-bonded region where parylene C (Cl in red color) was sandwiched between 2 PDMS membranes (Si in cyan color). Scale bars are 50 μm.

The USBD was fabricated based on selective bonding between 2 PDMS membranes with the help of a parylene C intermediate layer as shown in Fig. 1A. Basically, we made a membrane-type device in a 2D plane and then injected fluid to turn the 2D membrane-type device into a 3D balloon-type reservoir device, as shown in Fig. 2A. We chose PDMS and parylene C due to their highest biocompatibility, high flexibility, low stiffness, and mechanical properties close to those of biological tissues [33,34]. Selective bonding was achieved by activating the PDMS layer and the parylene C patterned PDMS layer by plasma oxidation, which is a standard method to create a silanol functional group (−OH) at the end of methyl groups (−CH_3_) for bonding and other surface functionalization [35–37]. After treating both layers with oxygen plasma, the monomer O–Si (CH_3_)_2_ of PDMS near the surface was converted into the hydroxyl group (–OH), ready for covalent bonding to another plasma-treated PDMS membrane. Thus, irreversible selective bonding was created between the PDMS layers, whereas the sandwiched parylene C area remained non-bonded as shown in Fig. 3A.

For cross-sectional analysis of the USBD, SEM-EDS was utilized as shown in Fig. 3B. The 2 PDMS layers and sandwiched parylene C were mapped based on Si and Cl atoms (Fig. S2), respectively. We could certainly distinguish the first PDMS layer (Si atoms), the intermediate parylene-C layer (Cl atoms), and the second PDMS layer (Si-atoms) through SEM-EDS images. Later, upon fluid injection, the non-bonded area turned into the inlet channel and the reservoir to enclose the RB solution (Fig. 2A). The RB solution in the reservoir could be released unidirectionally in a sustained manner (Fig. S3). The reservoir of the developed USBD was circular in shape (5 mm in diameter), with the device dimensions of 7 × 16 × 0.25 mm (width × length × thickness) before RB injection, and later turned into a balloon-like structure after RB injection, with dimensions of 7 × 16 × 2.5 mm. The reproducible drug loading capacity of each USBD was examined based on PDMS membranes with different compositions and thicknesses, ranging from 26 to 28 μl (Table S1). The size of the device is primarily dependent on the volume of the reservoir, which can be made smaller or larger enough to contain the amount of drug solution needed for the targeted therapy.

### In vitro release

We fabricated USBDs with various PDMS membrane thicknesses and compositions to assess different release kinetic profiles. To achieve release kinetic profiles based on the PDMS membrane composition, we solely relied on the mixing ratio of PDMS. PDMS is usually prepared as a cross-linked network of the base polymer and curing agent, and changing the mixing ratio of these 2 components changes the resulting density and molecular weight of the polymer chains between the adjacent cross-links [6,38,39]. Consequently, the cross-linking density based on the mixing ratio facilitated the controlled release of RB via diffusion. To evaluate the PDMS membrane based on compositions, the membrane thickness (178 μm), balloon diameter (5 mm), and RB concentration (0.5 w/v%) were kept constant for reproducible results. Similarly, another set of release kinetic profiles was achieved based on the changes in membrane thickness while keeping the PDMS membrane composition (10:1), balloon diameter (5 mm), and RB concentration (0.5 w/v%) constant according to Fick’s diffusion law [40].

#### Release kinetic profiles based on PDMS membrane thickness

**Fig. 4.**
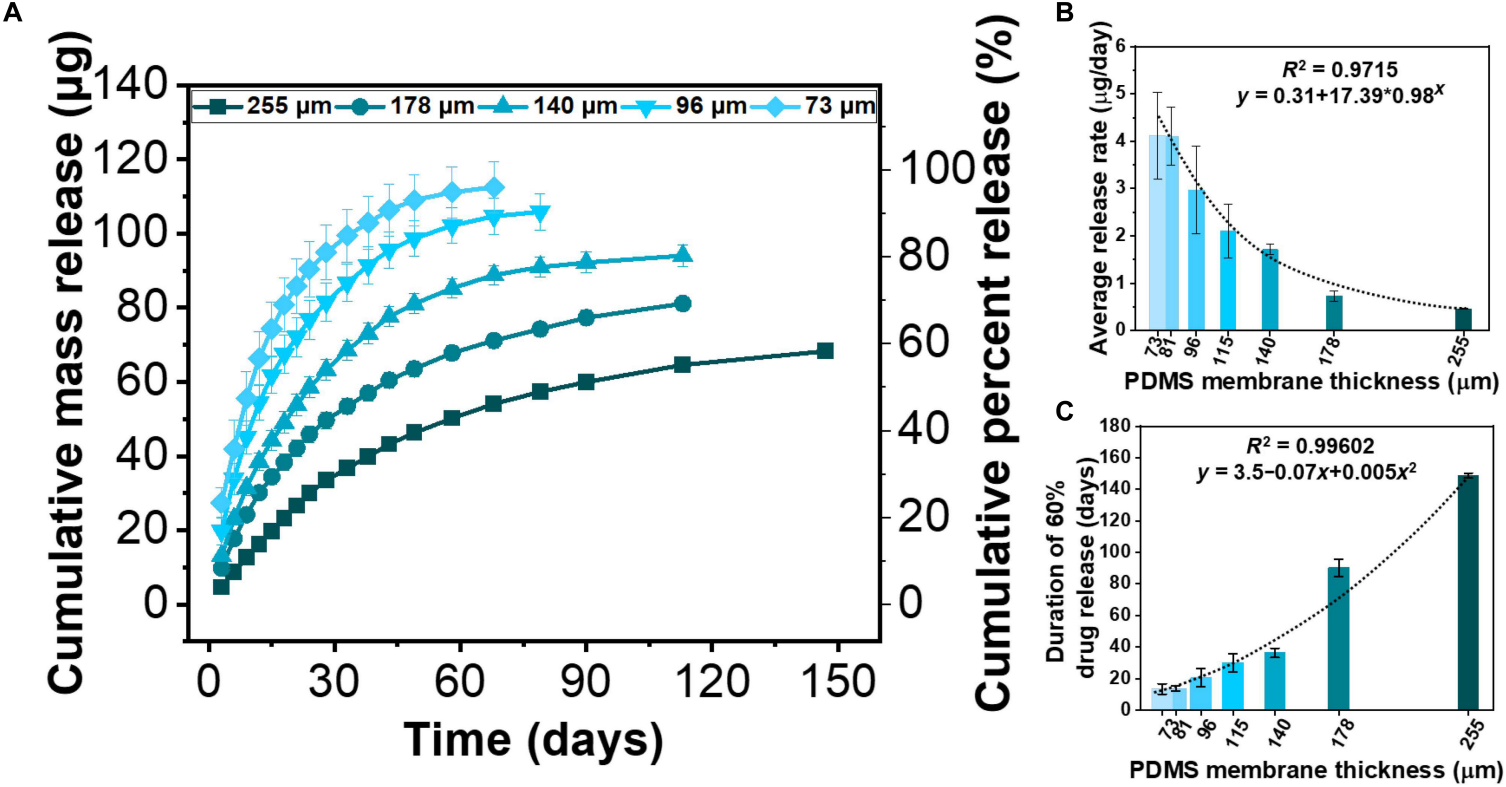
In vitro release kinetic profiles of USBDs based on PDMS membrane thickness: (A) effect of PDMS membrane thickness on the release kinetics, (B) average release rate to deliver 60% of the total amount of RB, and (C) duration of time to release 60% of the total encapsulated amount (*n* = 4; mean ± standard deviation).

Figure 4A depicts various release kinetic profiles based on PDMS membrane thicknesses. Drug release from 0% to 60% was considered in the determination of uniform slopes of all the profiles. The results indicate that the average release rate significantly increased non-linearly from 0.458 ± 0.008 μg/day to 4.11 ± 0.91 μg/day as the membrane thickness decreased from 255 μm to 73 μm, respectively (Fig. 4B). It is understandable that the drug release rate increases as the PDMS membrane thickness decreases since the drug diffusion distance becomes shorter [41]. The time needed to release 60% of the total amount was significantly increased non-linearly from 13.5 ± 3.31 days to 148.6 ± 1.5 days with increasing PDMS membrane thickness from 73 μm to 255 μm (Fig. 4C). In addition, the increase in release rate was not significant when the membrane thickness was increased from 73 μm to 96 μm. However, when the membrane thickness was increased beyond 96 μm, a significant change in the release rate was observed (Fig. 4B).

We also determined the longest period of zero-order release and nearly zero-order release under the conditions in which the coefficient of determination was *R*_1_^2^ > 0.99 and *R*_2_^2^ > 0.96, respectively, as shown in Fig. S4A and Table S2. Our findings revealed that regardless of the PDMS membrane thickness used, each USBD exhibited a zero-order release. Under these strict conditions, the longest period of zero-order release was achieved with USBD255 for 30 days (*R*_1_^2^ = 0.996) releasing 28.51% (28.51 μg) of the total amount with a release rate of 1.16 μg/day. Conversely, USBD73 showed the shortest zero-order release period of 15 days releasing 63.41% (74.32 μg) of RB with a release rate of 4.251 μg/day. Additionally, the longest period of nearly zero-order release was achieved up to 58 days (*R*_2_^2^ = 0.960) using USBD255 with a release rate of 0.827 μg/day, as shown in Table S2.

#### Release kinetic profiles based on PDMS membrane composition

**Fig. 5.**
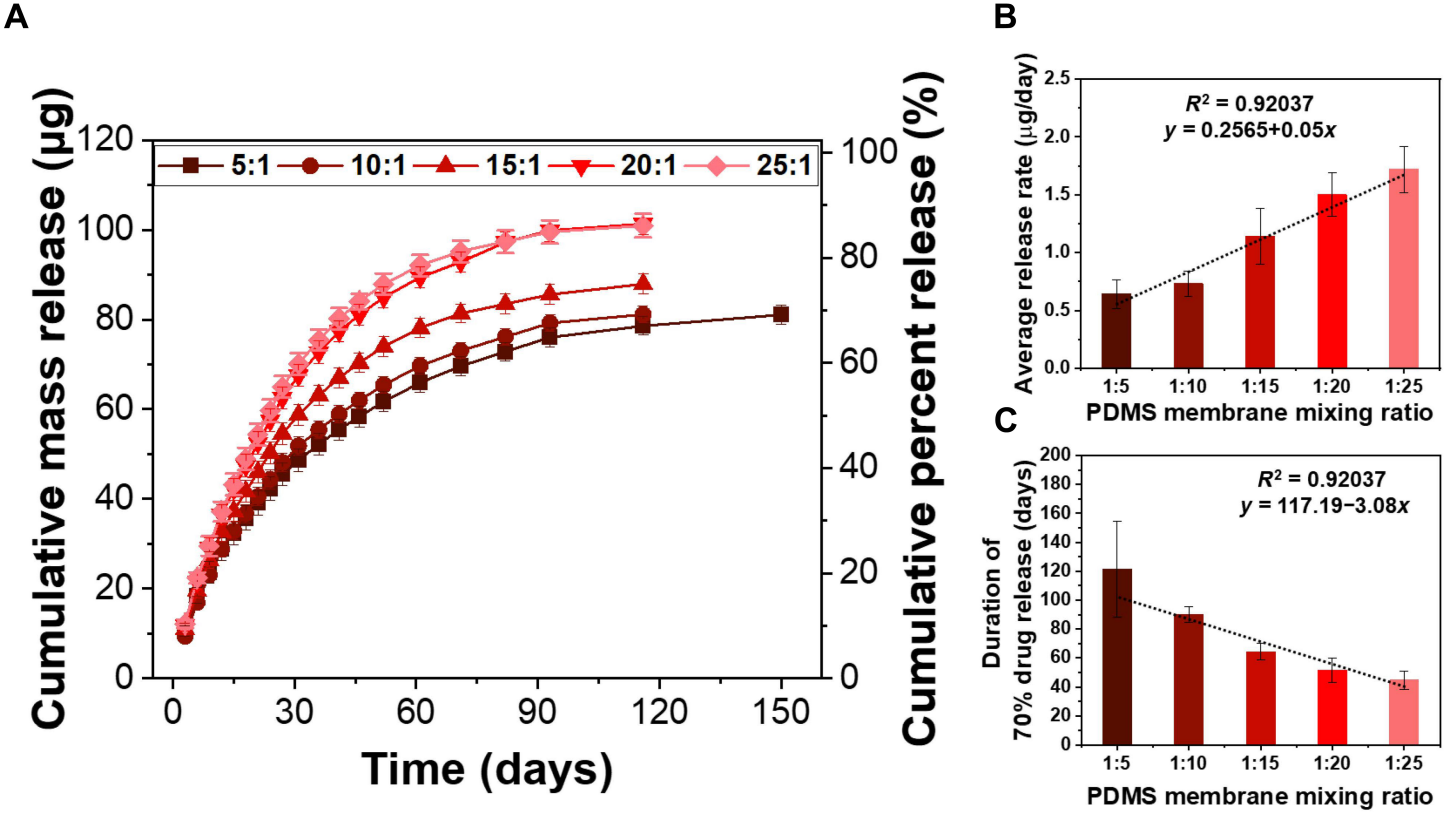
In vitro release kinetic profiles of USBDs based on PDMS membrane composition: (A) effect of PDMS composition on the release kinetics, (B) average release rate to deliver 70% of the total amount of RB, and (C) duration of time to release 70% of the total encapsulated amount (*n* = 4; mean ± standard deviation).

The effect of the PDMS membrane composition with different stoichiometric ratios on the release kinetics was investigated, as shown in Fig. 5A. Drug release from 0% to 70% was considered in the determination of uniform slopes of all the profiles. The average release rate linearly increased from 0.642 ± 0.125 μg/day to 1.721 ± 0.19 μg/day when the amount of cross-linking agent decreased from 1:5 (USBD5) to 1:25 (USBD25), respectively (Fig. 5B). When the amount of curing agent decreases, the molecular weight between the adjacent cross-links increases as opposed to the cross-linking density, and as a result, the membrane swelling properties and permeability increase, facilitating a higher release rate. The effect was also dominant on the total release time to release 70% of the total amount. The release time linearly increased from 44.6 ± 6.35 days to 127.25 ± 27.89 days when the curing agent ratio was decreased from 1:5 to 1:25, respectively (Fig. 5C). Regardless of the PDMS membrane composition, each device exhibited zero-order release. The longest zero-order release was achieved with USBD25, where the lowest amount of curing agent was used, for 21 days (*R*_1_^2^ = 0.992), releasing 46.34% (54.31 μg) of the total amount with a release rate of 2.431 μg/day. In contrast, USBD5 (1:5) exhibited the shortest zero-order release period of 12 days, releasing 24.57% (28.8 μg) of RB with a release rate of 2 μg/day. In addition, the longest nearly zero-order release was achieved for 52 days (*R*_2_^2^ = 0.967) through USBD25 (Fig. S4B and Table S2), with a release rate of 1.71 μg/day. It is evident that the USBD can be customized with various membrane compositions to achieve various release kinetic profiles.

#### In vitro cell viability

**Fig. 6.**
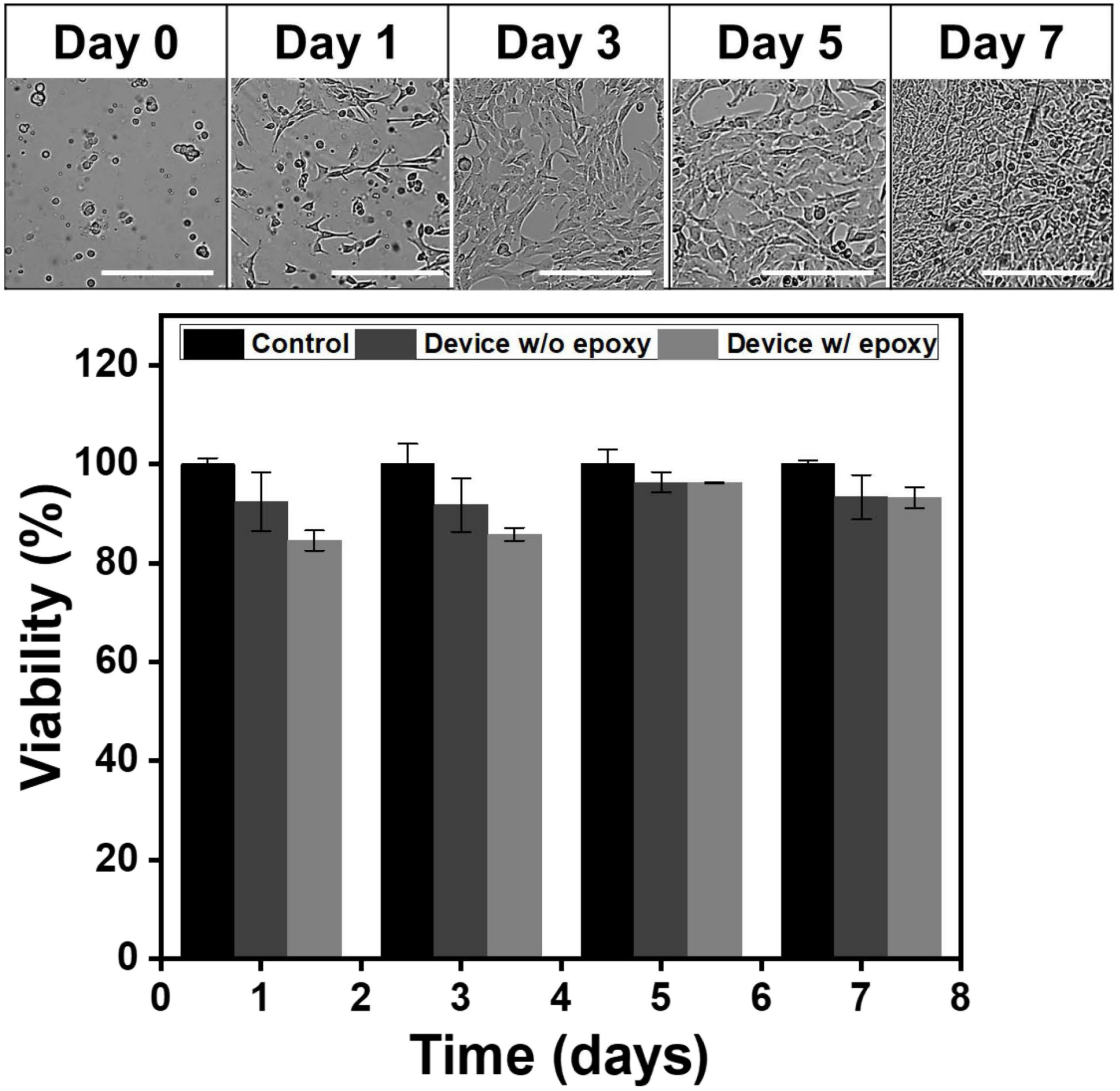
Evaluation of cell viability of the USBD using C2C12 myoblast cells. Cell proliferation on USBDs with silicone epoxy over time is shown on top (*n* = 4; mean ± standard deviation). Difference in cell viability between the groups without and with silicone epoxy was not significant. Scale bars are 200 μm.

When tested with C_2_C_12_ mouse myoblast cells, USBDs showed excellent biocompatibility as demonstrated in Fig. 6. The cell viability was investigated with 2 groups including USBDs without and with silicone epoxy, and the resulting average cell viability was maintained ≥90% for 7 days in both groups. Silicone epoxy (Kwik-Cast) has been frequently used with implantable devices mainly for device fixation as it has excellent biocompatibility [42–44]. The USBDs with silicone epoxy showed lower cell viability relative to the USBDs without silicone epoxy during the first 3 days but later reached more than 90%. This might have happened due to poor cell adhesion to the device surface or cell adjustment to the environment during the first few days. However, there was no significant difference in cell viability between the 2 groups. These results demonstrate that the developed USBD did not exhibit cytotoxic effects.

### In vivo evaluation

**Fig. 7.**
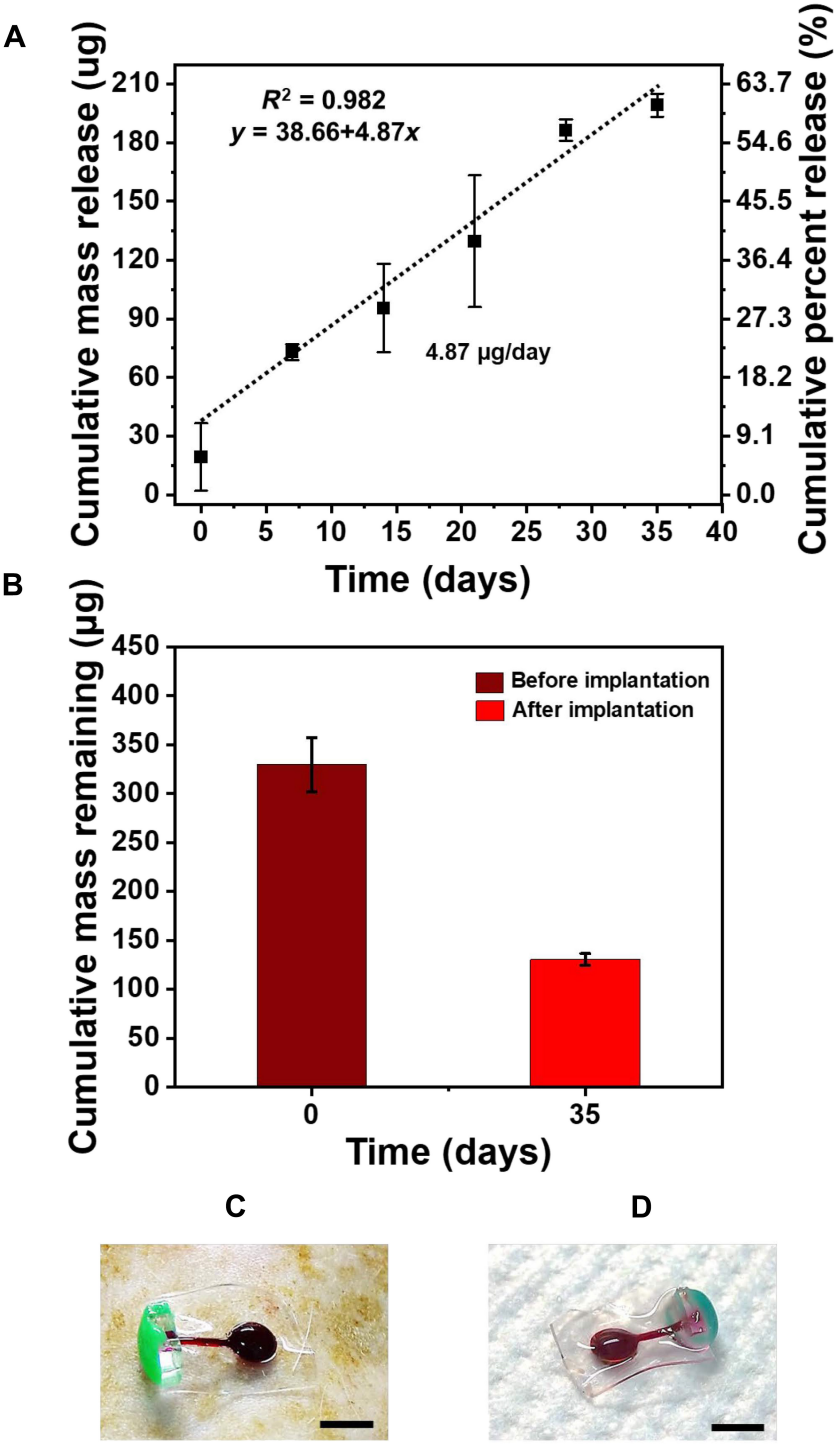
In vivo pharmacokinetics of the USBD: (A) in vivo drug release characteristics of a representative USBD (USBD81) for 35 days, (B) remaining RB in USBD before implantation and after explantation at 35 days. During implantation for 35 days, 60.42% of the total amount was released. Optical images of USBD (C) before implantation and (D) after explantation. Scale bars are 5 mm.

To assess the in vivo performance of the developed USBD, pharmacokinetic evaluation was performed for 35 days (Fig. 7A). USBD81 was implanted in rats and was easily removed from the implantation site at the end of the experiment (Fig. 7C and D). In vivo drug release kinetics are usually investigated by measuring the drug concentration in the plasma of animals implanted with drug delivery devices [11,45]. However, this method is limited by drug loss due to systematic effects such as hepatic metabolism and renal and biliary elimination clearance [46]. Therefore, the changes in RB amount inside the reservoir were quantified by measuring the residual amount of RB in the explanted USBD. To demonstrate the in vivo release of RB, USBD81 was explanted (*n* = 3) at scheduled intervals to extract the residual amount of RB in each USBD, and the released RB amount was later calculated compared to non-implanted devices (baseline), as shown in Fig. 7A. In the in vivo environment, USBDs exhibited zero-order release for up to 28 days (*R*^2^ = 0.995), releasing 56.5% (186.41 μg) of the total amount with a release rate of 5.4 μg/day. We also noticed a nearly zero-order release (*R*^2^ = 0.981) during the whole period of in vivo evaluation, releasing 60.4% (199.11 μg) of the total amount while maintaining a release rate of 4.87 μg/day. The amount of RB in USBD’s reservoir was compared before and after implantation as shown in Fig. 7B. These findings clearly demonstrate that the in vivo release characteristics of the USBD were consistent with the in vitro release kinetics. Here, the zero-order release characteristics were maintained for up to 28 days, as demonstrated in the in vitro evaluation (Fig. S5).

#### In vivo biodistribution using IVIS imaging

**Fig. 8.**
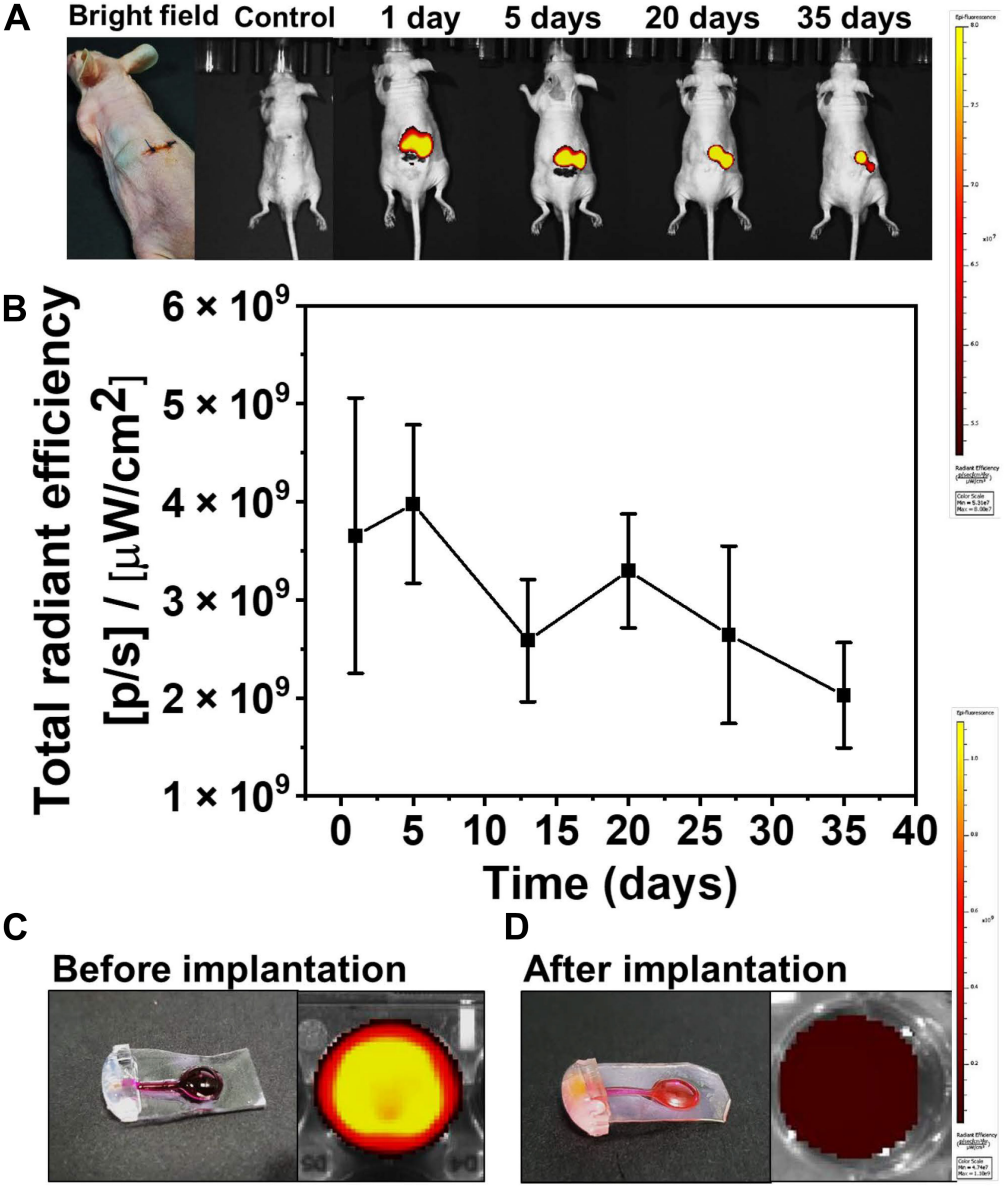
In vivo biodistribution of RB: (A) Representative IVIS fluorescence images of the USBD implanted subcutaneously in living nude mice (BALB/c). (B) Quantitative total radiant efficiency of the implanted USBD showing a decrease in signal intensity over time. (C and D) Bright-field image of the USBD (left) and initial fluorescence intensity of RB in the USBD (right) (C) before implantation and (D) after implantation. Color bars on the right represent the signal intensity.

To further investigate the in vivo performance of USBDs, we implanted USBDs (*n* = 3) containing RB as a model drug in BALB/c nude mice. The fluorescence intensity of the implanted USBD was visualized at pre-determined time intervals using IVIS. In the experimental group, the localized fluorescence signal was detected only near the implanted USBD, as shown in Fig. 8A. As the sustained release of RB continued from the implanted USBD, fluorescence intensity gradually decreased over time due to a decline in the RB concentration within the USBD reservoir (Fig. 8B). The localized fluorescence signal was continuously detected for up to 55 days. However, the localized signal intensity remained nearly constant after 35 days, mainly due to the fibrotic capsule surrounding the USBD. A further decrease in the IVIS signal may be noticed once the fibrotic capsule stabilizes over time. Afterwards, the USBDs were explanted at 55 days, and clearly, the RB concentration was significantly changed in the device before and after implantation, demonstrating a sustained release of RB, as shown in Fig. 8C and D. These results further validated the in vivo performance of the developed USBD.

#### Histopathological evaluation

**Fig. 9.**
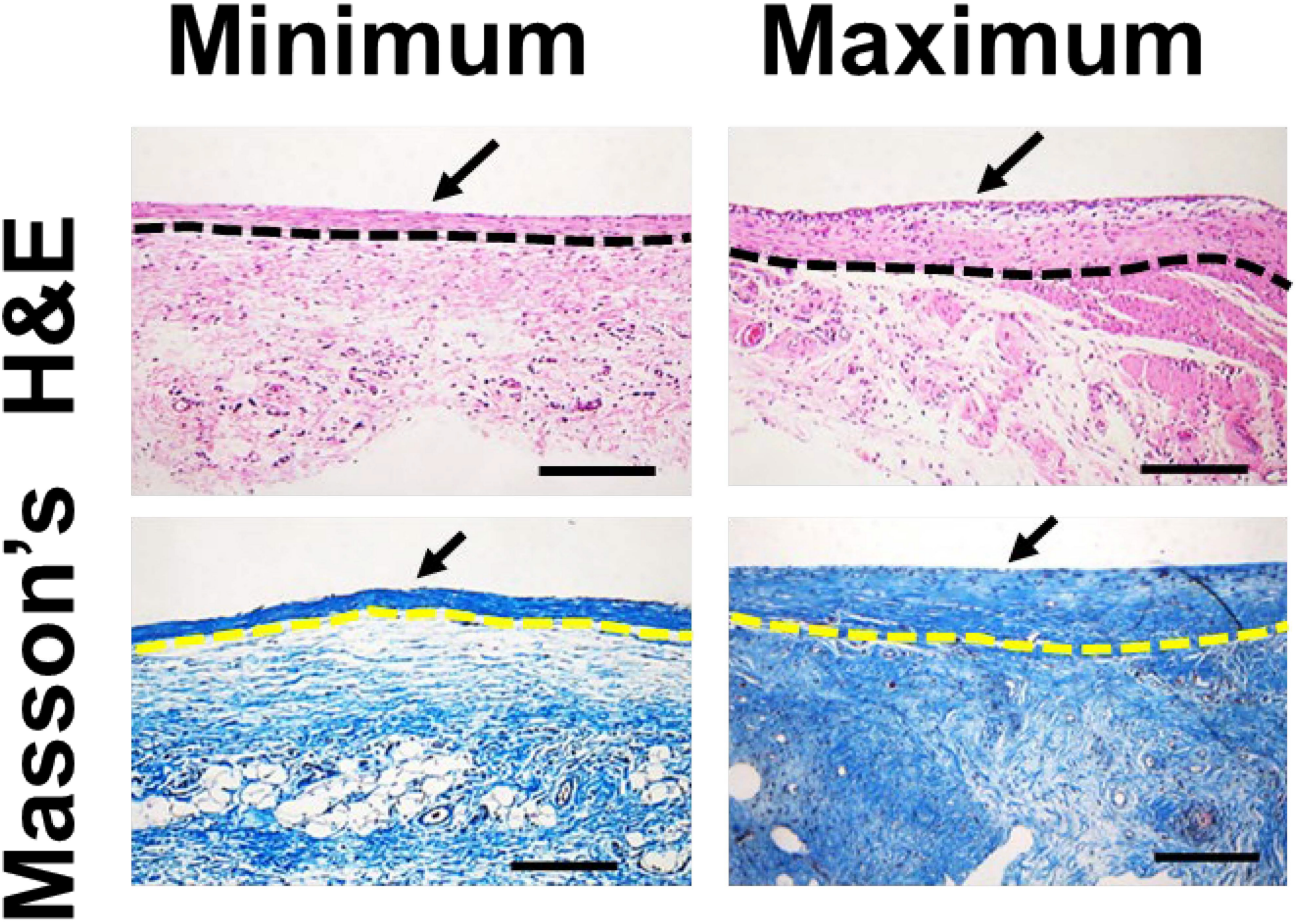
Representative histological images of the tissues where USBDs were implanted for 35 days (*n* = 4). Histological sections show the fibrotic capsule thickness, marked by black and yellow dotted lines. The minimum and maximum capsule thicknesses were 53.33 ± 16.02 μm and 174.18 ±63.10 μm, respectively. The boundary of the device is indicated by black arrows. Scale bars are 200 μm.

Tissues surrounding the USBDs were harvested 35 days after implantation at the end of the experiment for histological evaluation (Fig. 9). The groups of animals (*n* = 4) implanted with USBDs did not exhibit any complications as demonstrated in the biopsied tissues (Fig. 9). However, H&E and Masson’s trichrome staining of the tissues surrounding the USBDs confirmed the formation of fibrotic capsule as shown in Fig. 9. The minimum, maximum, and average capsule thicknesses found around the USBD, at different locations such as inlet channel, reservoir, and bonded PDMS membranes, were 53.33 ± 16.02 μm, 174.18 ±63.10 μm, and 112.64 ± 56.08 μm, respectively.

## Discussion

Implantable devices for controlled release have been gaining attention for localized long-term drug delivery [7]. However, their performance is limited by high initial burst, risk of membrane rupture because of in vivo degradation, high onset time, non-conformal contact with biological tissues, tissue damage, and excessive foreign body responses. Hence, there has been difficulty in achieving accurately controlled drug delivery. Previously, the only soft implantable device embedded with micro-channels to realize almost zero-order release was reported in [11], which can be folded to reduce the size of the incision during surgery. Channels in this soft device were connected in parallel to the drug reservoir, where the drug release rate was modulated by varying the channel area and length. However, the releasing channels were laterally distributed on the sides of the device; therefore, direct contact with target tissues after implantation was not possible, which limits the aim of targeted drug delivery and could cause off-target toxicity. Zero-order release was achieved using the longest channel, which inevitably increased the device size, making the surgical procedure more invasive during explantation. The device was not sufficiently flexible to be retracted from the subcutaneous pocket in folded form without causing extensive tissue damage. Moreover, using drugs in powder form and then diffusion of drugs through long channels increased the onset time, which could cause an excessive delay in drug release. Although the device was made of soft materials using molding techniques, the device was relatively hard and rigid due to the thickness of the device, which resulted in high foreign body responses with a minimum capsule thickness of 858.818 ± 52.6 μm. Conversely, rigid-type devices, including micro-chips embedded with micro-channels, were also used to achieve almost zero-order release [12,47]. These micro-chips were fabricated using rigid materials, resulting in severe foreign body responses with a fibrotic capsule thickness ranging from 903.9 ± 48.91 to 990.9 ± 111.5 μm. In addition, the lateral release channel, mainly by modulating the release rate, can cause off-target toxicity and increase the device size, which is less favorable for implantation. The USBD developed in our study shows superior characteristics to overcome the aforementioned limitations in the previous devices, as explained in detail as below.

### Minimized membrane rupture, initial burst, and onset time

The developed ultrasoft balloon-type structure, without sharp and rigid edges, achieved a high degree of softness, flexibility, and stretchability as shown in Fig. 2B. Owing to the optimized fabrication process of irreversible selective bonding and intrinsic inertness of PDMS, the fabricated USBDs did not show any membrane rupture during the time of characterization and release study for more than 5 months, and could be easily retrieved after the implantation period with a small incision (Fig. S7). We injected RB solution into the USBD reservoir instead of RB powder, which inherently reduced the onset time and started immediate release after implantation, as shown in Fig. S6. During the in vitro evaluation, an initial burst was not observed in any of the experimental groups, and all devices remained intact during the evaluation period of 5 months. In addition, the USBD in 2D form can be easily transferred in bulk quantity from one place to another with minimum care and expenses; later, it needs a simple and less spacious setup at the target site of implantation (syringe pump, syringe, silicone tube, and silicone adhesive) to inject the therapeutic solution into the reservoir (Fig. S8).

### Zero-order release in in vitro and in vivo environments

Our findings revealed that regardless of the PDMS membrane thickness and composition, each USBD exhibited zero-order release. By varying the membrane thickness and composition, we could precisely control the release rate, release time, total amount of RB release, and release type. The longest period of zero-order release was achieved with USBD255 for 30 days (*R*_1_^2^ = 0.996) with a release rate of 1.16 μg/day. Additionally, the longest period of nearly zero-order release was achieved for up to 58 days (*R*_2_^2^ = 0.960) using USBD255, with a release rate of 0.827 μg/day, as shown in Table S2.

The in vivo release characteristics of USBDs were consistent with the in vitro release kinetics. The zero-order in vivo release characteristics were maintained for up to 28 days, as demonstrated in the in vitro evaluation (Fig. S5). However, there was an increase in the release rate in in vivo conditions, probably due to the complex biological environment with diverse components working as surfactants in the biological fluid [48]. Moreover, the RB release can be affected by in vivo environmental changes and a high clearance rate, which may have sustained a high concentration gradient, hence expediting RB out-diffusion [49]. In addition, the zero-order release time was slightly reduced in the in vivo environment, mainly due to fibrotic capsule formation, which is inevitable for many nondegradable implantable devices [50,51]. However, the zero-order release period achieved by our device was higher than that of previously developed devices [11,12,47], mainly due to the device compatibility with biological tissues and delayed fibroblast encapsulation, which resulted in prolonged zero-order release in the in vivo environment.

### Minimized foreign body responses due to ultrasoft mechanical properties

The degree of inflammation around the USBD was assessed to be minimal and noticeably less than that of previously developed devices [11,12,47,52]. Ji et al. [11,12,47] developed devices with a minimum fibrotic capsule thickness from 858.818 ± 52.6 μm to 990.9 ± 111.5 μm after 30 days of implantation. Similarly, Bose et al. [52] reported a capsule thickness ranging from 150 to 250 μm after 28 days of implantation. Evidently, the capsule thickness of the developed USBD was significantly lower than that of abovementioned devices. The minimum capsule thickness was ascribed to the flexibility and biocompatibility of PDMS and parylene C, the main constituent materials of the developed device [34]. Both PDMS and parylene C have demonstrated excellent long-term biocompatibility, biodurability, and in vivo stability. Their ability to maintain structural integrity and no cytotoxicity for years within the biological environment qualifies them as ideal candidates for chronic implantable devices, encompassing a broad range of therapeutic and diagnostic applications [25–29]. The pros and cons of the materials used in the USBD and previously developed devices are summarized in Table S3. In addition, chronic foreign body responses are tied to tissue trauma during and after implantation caused by the device design and mechanical properties [19]. An optimized design with spherical and balloon shape along with ultrasoft mechanical properties of the used materials can reduce foreign body responses [53–55]. Therefore, the minimum foreign body responses achieved in our study were attributed to the balloon-type device design with no sharp edges and the inherited ultrasoft mechanical properties preventing subsequent trauma around the tissue. The histopathological results of the USBD demonstrated excellent biocompatibility in the in vivo environment with minimal foreign body responses. Noticeably, the selected USBD materials, shape, and ultrasoft mechanical properties have proven to be effective in reducing the foreign body responses and prolonging the sustained release. However, further study is needed to elucidate the factors influencing long-term biocompatibility and biological tissue interaction with the USBD to understand how the fibrotic capsule thickness varies over time and its effects on the drug delivery efficacy during long-term implantation.

Since the USBD was presented as a proof of concept, we used RB as a releasing agent to better understand and visualize the in vivo and in vitro release kinetic characteristics before adapting a disease model. Therefore, our study lacks a disease model to investigate the efficacy of the device in delivering clinical drugs. We plan to validate the device with a glioblastoma disease model using potent anti-cancer clinical drugs, such as doxorubicin (MW: 543.5 g/mol) and diclofenac sodium (MW: 296.1 g/mol). Both drugs have molecular weights similar to that of RB [56,57]. Multiple clinical drugs can also be delivered using multi-reservoir-based devices utilizing customized PDMS membranes with modified physical and chemical characteristics specific to the chosen drug’s chemical and physical properties. Based on the molecular weight and the release rate of the target drugs, the porosity of the PDMS membrane can be modified using leaching techniques that can facilitate release [58,59]. It is expected that the developed device can be implemented with a variety of cancer disease models, such as subcutaneous, eye, and brain surface lesions.

### Conclusion

We developed an ultrasoft and flexible balloon-type implantable drug delivery device with distinct release kinetics for long-term controlled release. The suggested device design could achieve immediate onset and unidirectional controlled release. By modulating the PDMS membrane thickness and composition, we demonstrated zero-order and nearly zero-order releases without initial burst release, and various release kinetics profiles were achieved. Most importantly, the ultrasoft mechanical properties of the device ensured less foreign body responses than previously developed reservoir-type devices, with relatively thin fibrotic encapsulation while minimizing tissue damage. Additionally, the device exhibited and maintained zero-order and nearly zero-order releases in in vivo environments. We anticipate that localized delivery using the developed USBD may facilitate the administration of drugs that would not be suitable for systematic use due to side effects and could be a promising technique to achieve long-term zero-order release.

### Ethics approval and consent to participate

All in vivo experiments were conducted following the experimental protocols approved by the Institutional Animal Care and Use Committee (Approval No. DGIST-IACUC 23012602-0004) at DGIST.

## Data Availability

All data generated or analyzed during this study are included in this published article or the Supplementary Materials.
