# Erratum to “An Ultrasoft and Flexible PDMS-Based Balloon-Type Implantable Device for Controlled Drug Delivery”

**DOI:** 10.34133/bmr.0126

**Published:** 2024-12-23

**Authors:** Tausif Muhammad, ByungWook Park, Aseer Intisar, Minseok S. Kim, Jin-Kyu Park, Sohee Kim

**Affiliations:** ^1^ Department of Robotics and Mechatronics Engineering, Daegu Gyeongbuk Institute of Science and Technology, Daegu 42988, Republic of Korea.; ^2^ Department of New Biology, Daegu Gyeongbuk Institute of Science and Technology, Daegu 42988, Republic of Korea.; ^3^ Department of Veterinary Pathology, College of Veterinary Medicine, Kyungpook National University, Daegu 41566, Republic of Korea.

In the Research Article “An Ultrasoft and Flexible PDMS-Based Balloon-Type Implantable Device for Controlled Drug Delivery,” the authors identified an error in the schematic illustration of the device [1]. Specifically, the bonded region in Figure 1A incorrectly showed a Parylene C layer (in yellow color) between the PDMS-PDMS bonding created using O₂ plasma treatment. However, in the actual device, this bonded region should not include a Parylene C layer. Figure 1 has now been corrected in the original publication, and the authors apologize for any confusion that this inadvertent error may have caused.
