# Supplementary material for: An Ultrasoft and Flexible PDMS-Based Balloon-Type Implantable Device for Controlled Drug Delivery
Source: Biomater Res. 2024 Mar 28;28:0012. doi: 10.34133/bmr.0012 (PMC10981933; doi:10.34133/bmr.0012)
Supplement: Supplementary 1 — Figs. S1 to S8 Tables S1 to S3 [file bmr.0012.f1.docx]

# SUPPLEMENTARY INFORMATION

# An ultra-soft and flexible PDMS-based balloon-type implantable device for controlled drug delivery

Tausif Muhammad^a^, Byungwook Park^a^, Aseer Intisar^b^, Minseok S. Kim^b^, Jin Kyu Park^c^, Sohee Kim^a, *^

^a^ Department of Robotics and Mechatronics Engineering, Daegu Gyeongbuk Institute of Science and Technology, Daegu 42988, Republic of Korea

^b^ Department of New Biology, Daegu Gyeongbuk Institute of Science and Technology, Daegu 42988, Republic of Korea

^c^ Department of Veterinary Pathology, College of Veterinary Medicine, Kyungpook National University, Daegu 41566, Republic of Korea

Corresponding author at: Department of Robotics and Mechatronics Engineering, Daegu Gyeongbuk Institute of Science and Technology, Daegu 42988, Republic of Korea. E-mail address: [soheekim@dgist.ac.kr](mailto:soheekim@dgist.ac.kr) (Sohee Kim)

**Figure S1.** Surgical procedure of USBD implantation. An USBD is inserted through a surgical incision less than the size of the device owing to its ultra-soft and flexible properties: (A) The dorsal area is shaved and sterilized with betadine, and a skin incision of 6mm is made for device implantation. (B) The USBD is inserted through the surgical incision into the subcutaneous pocket, (C) with a magnified view of it. (D) The incision is closed with a surgical suture and disinfected with betadine.


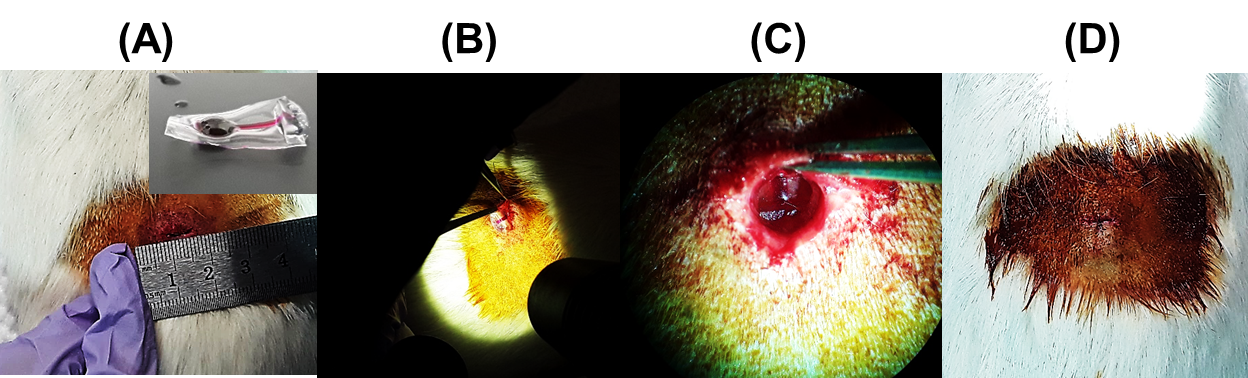

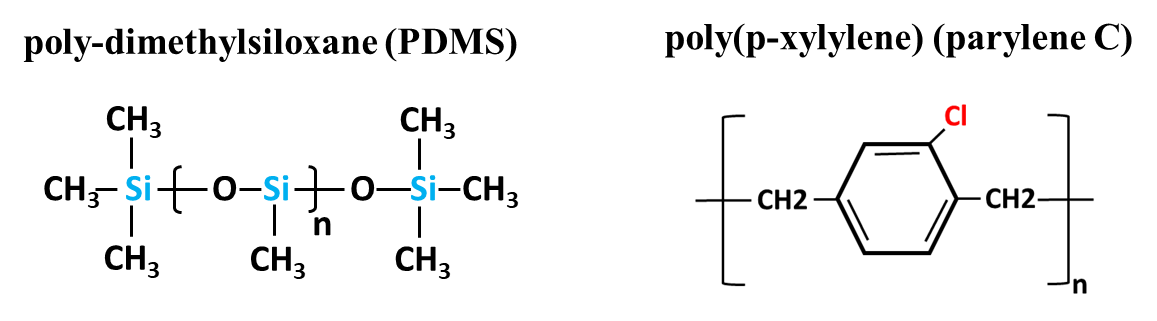


**Figure S2.** Chemical structures of PDMS and parylene C.

**Figure S3.** (A) Directional release of RB using the developed USBD. (a) The USBD was immersed in PBS for 25 days, (b) after 25 days, the reservoir was emptied, (c) USBD’s cross-section sandwiched between PDMS blocks, (d) then cross-section of the USBD was examined under fluorescence microscope. The bare PDMS membrane could release RB while the parylene C patterned PDMS membrane blocked the outflux of RB. (B) USBD releasing RB on agarose gel for 5 days. Here, a unidirectional release to avoid off-target toxicity was achieved.


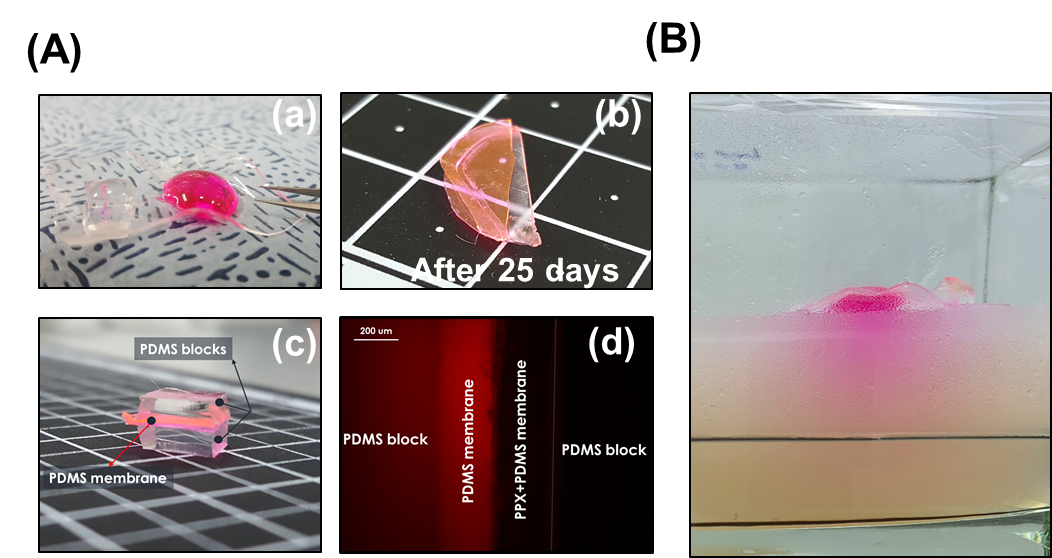


**Figure S4.** In vitro evaluation of zero-order (R_1_^2^ > 0.99) and nearly zero-order (R_2_^2^ > 0.96) release kinetics using mathematically predicted coefficients of determination based on (A) PDMS membrane thickness and (B) PDMS membrane composition.


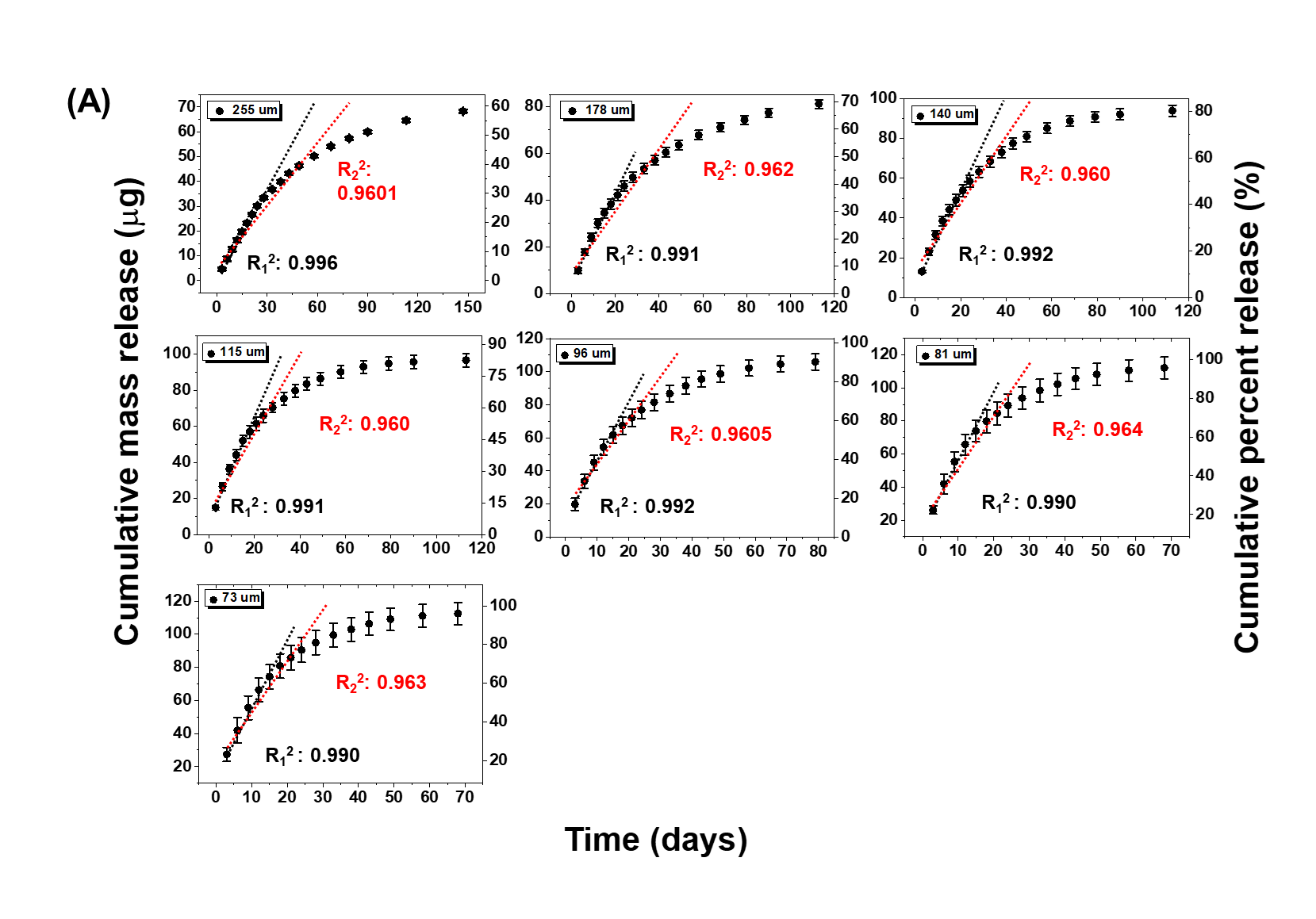

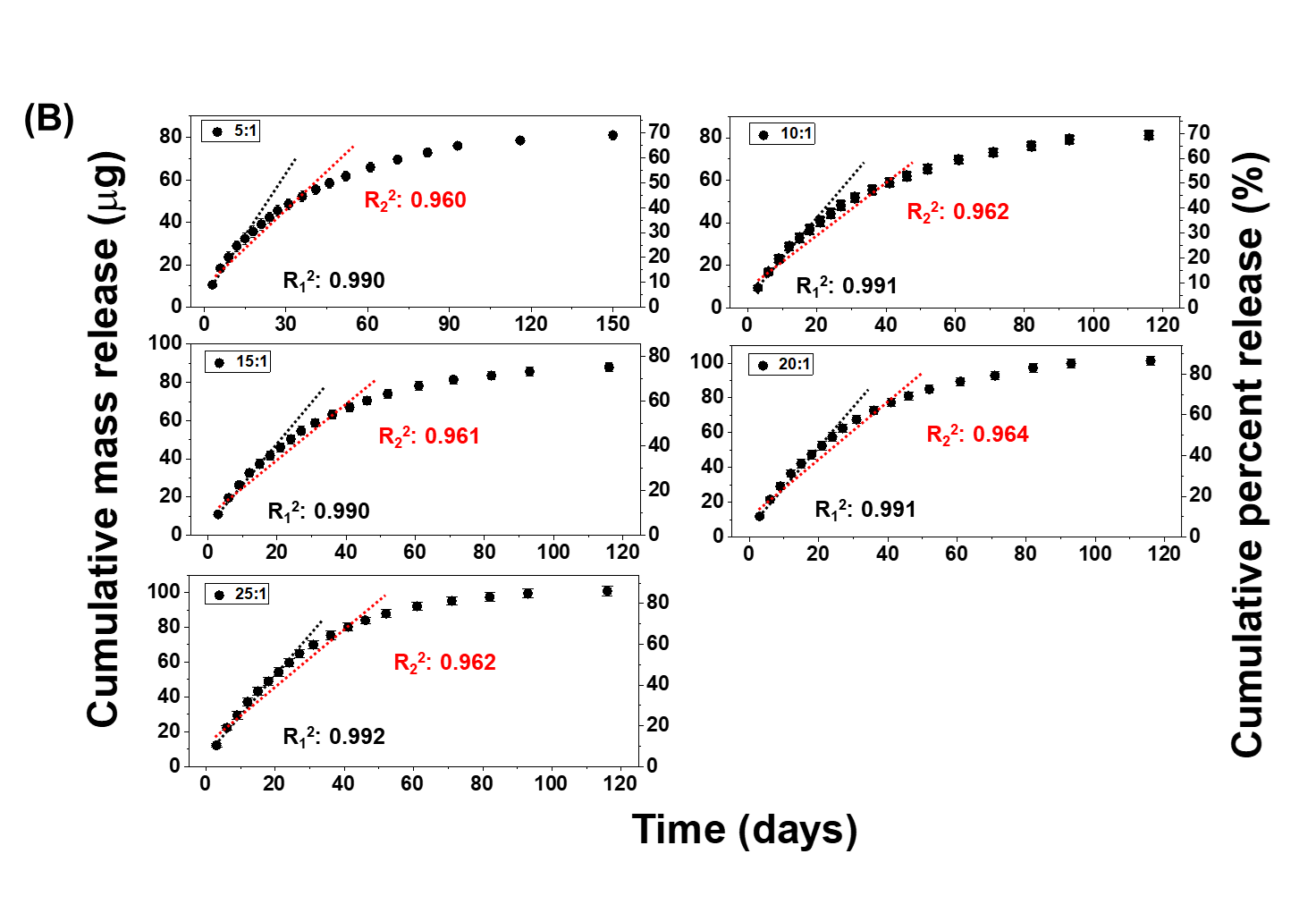


**Figure S4.** In vitro evaluation of zero-order (R_1_^2^ > 0.99) and nearly zero-order (R_2_^2^ > 0.96) release kinetics using mathematically predicted coefficients of determination based on (A) PDMS membrane thickness and (B) PDMS membrane composition.


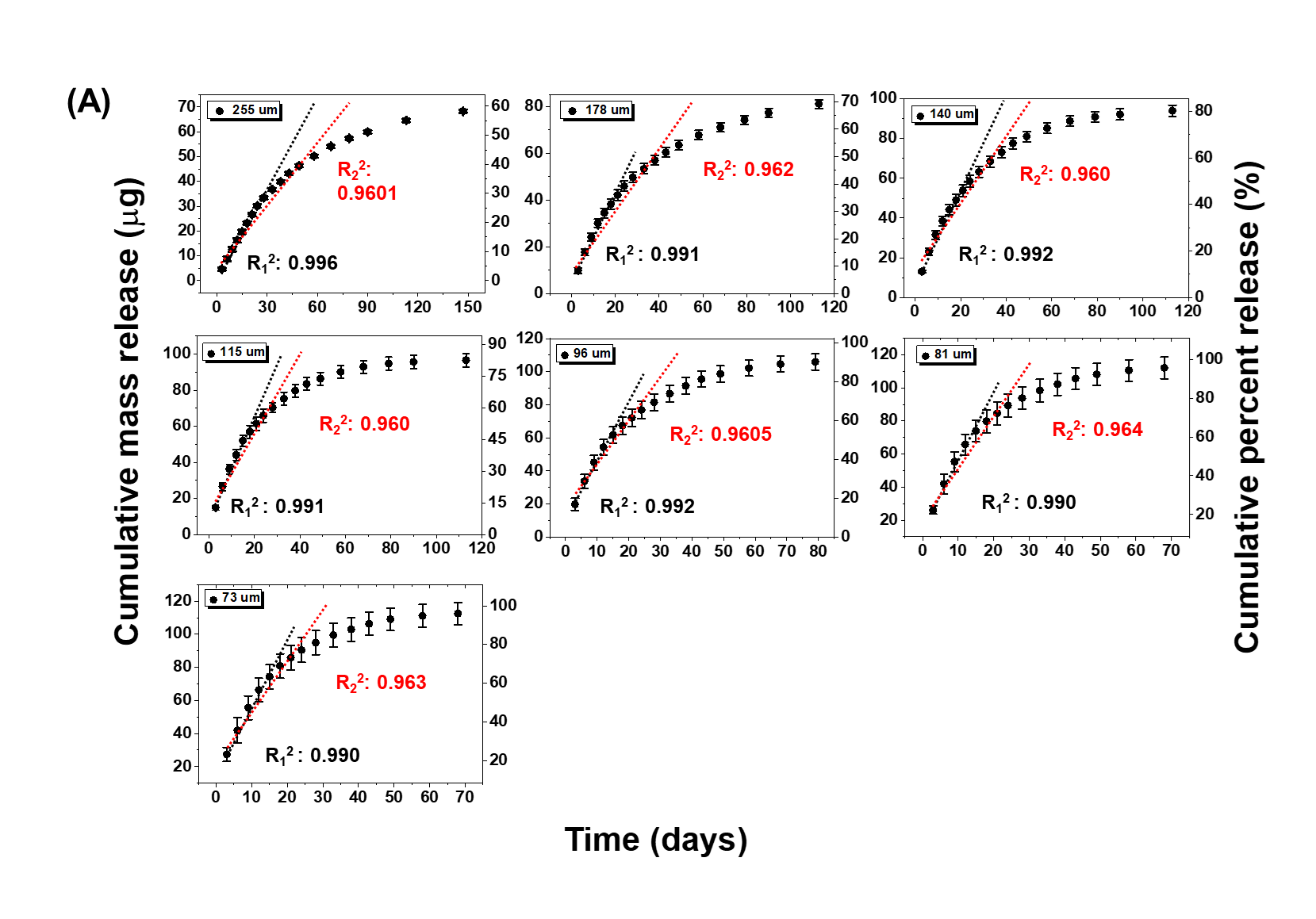

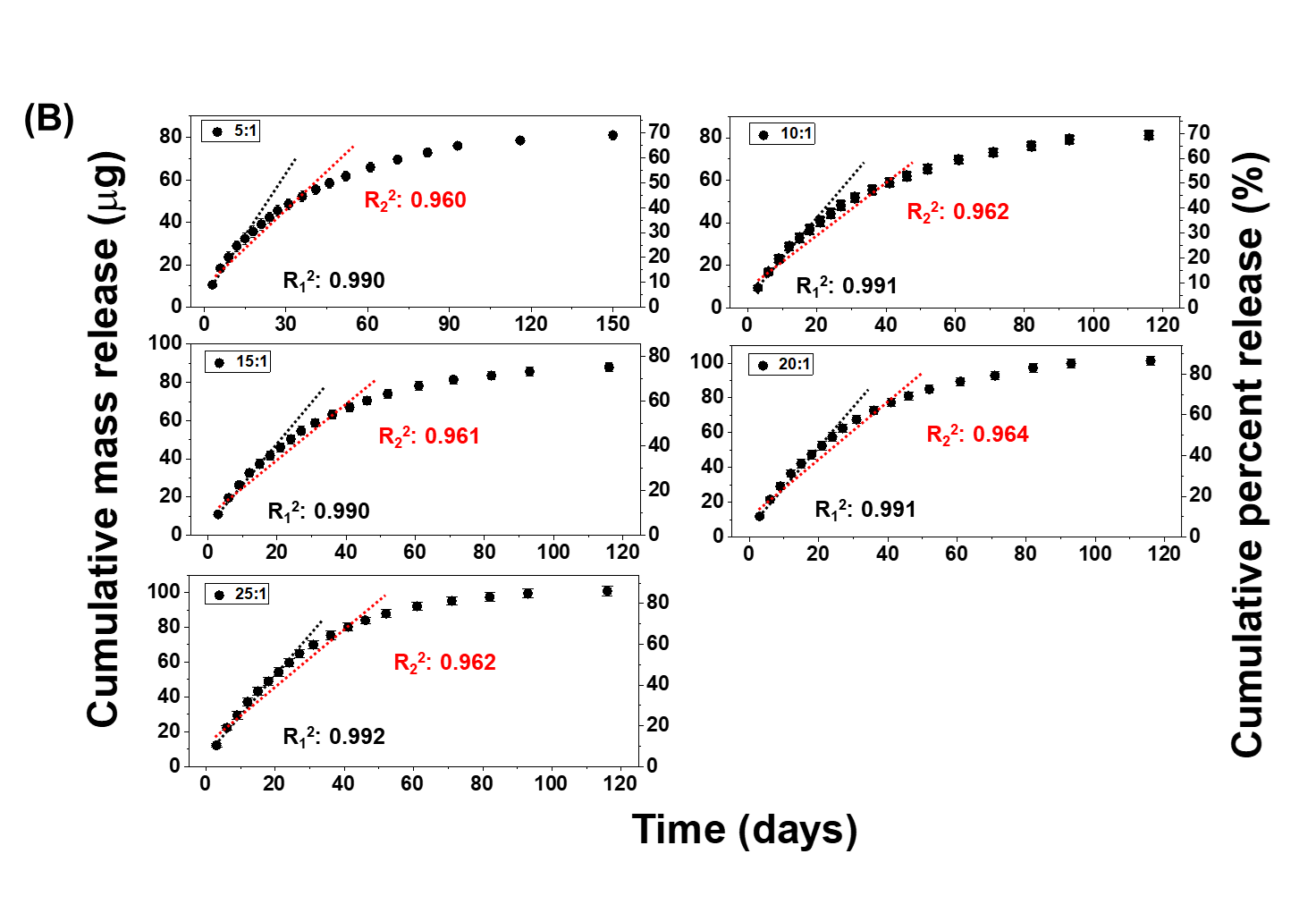


**Figure S5.** In vitro and in vivo release profiles of USBDs loaded with RB (n=4; mean ± standard deviation). For the analysis of in vitro release kinetics, we prepared 4 devices loaded with 1.5w/v% of RB and immersed in 20 ml of PBS at 37^o^C. The aliquots were collected at scheduled intervals for 35 days and measured spectrophotometrically at 551 nm using UV-Vis spectrophotometer. After collecting the aliquots, previously used PBS was replenished with fresh PBS to avoid RB saturation in the receiving phase. For the analysis of in vivo release kinetics, see Section 3.3. Our in vivo result revealed that the implanted USBDs continuously released drug in a pattern comparable to that shown in in vitro drug release profiles. However, there was an increase in the RB release rate for in vivo results, probably due to the complex biological environment with diverse components working as surfactant in the biological fluid [1]. Moreover, RB release can also be affected by in vivo environmental changes and high clearance rate of body fluids, which may have sustained a high concentration gradient, hence expediting RB out-diffusion [2].

**Figure S6.** Rhodamnine B penetration through a PDMS membrane with a thickness of 115 µm. A drop of Rhodamine B was left for 10 minutes on the PDMS membrane. Later, the membrane was washed with isopropanol and DI water, and the sliced samples were examined under fluorescence microscope. The PDMS membrane was sliced at two different locations marked by white dotted lines as slice (1) and (2), showing that Rhodamine B penetrated through the PDMS membrane in 10 minutes or less, regardless of the location. It confirms that immediate onset can be achieved after Rhodamine B injection into the reservoir of the USBD.


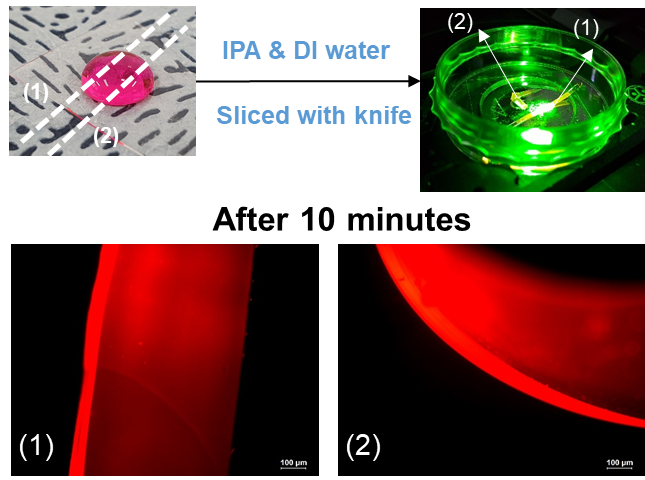


**
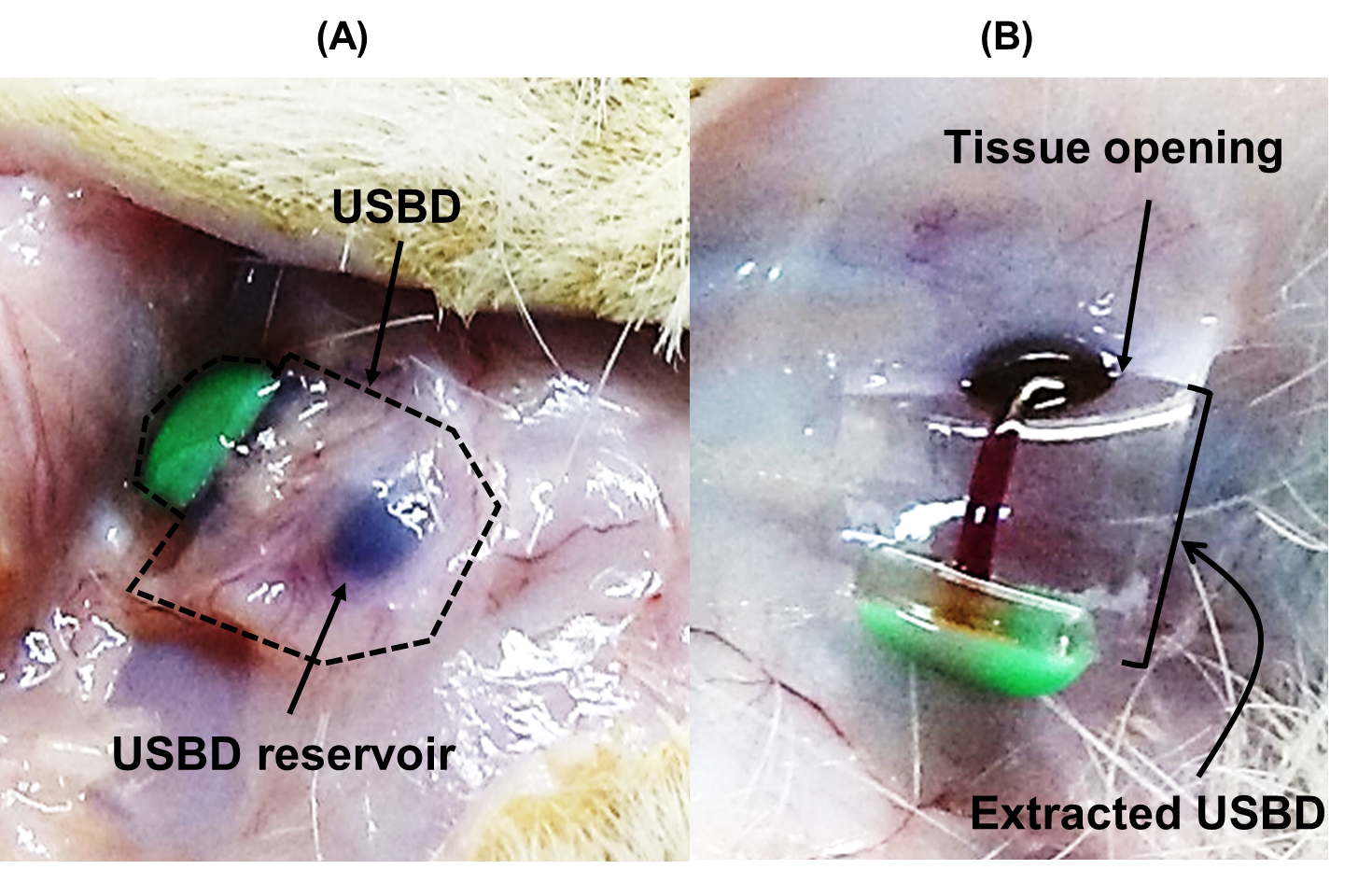
**

**Figure S7.** USBD explantation after 35 days from living rats (A) Tissue surrounding the USBD after skin incision. A large skin incision was made to clearly picture the USBD encapsulated by surrounding tissues (B) Taking out the USBD after a small incision from the surrounding tissue pocket.

**Figure S8.** Preparation of the USBD for implantation at the target site. After transporting the devices in bulk quantity from the fabrication site to the implantation site, only a small and compact setup is needed to prepare the USBD for implantation. RB solution is injected into the USBD’s reservoir using a syringe, silicone tube and syringe pump at the implant site.


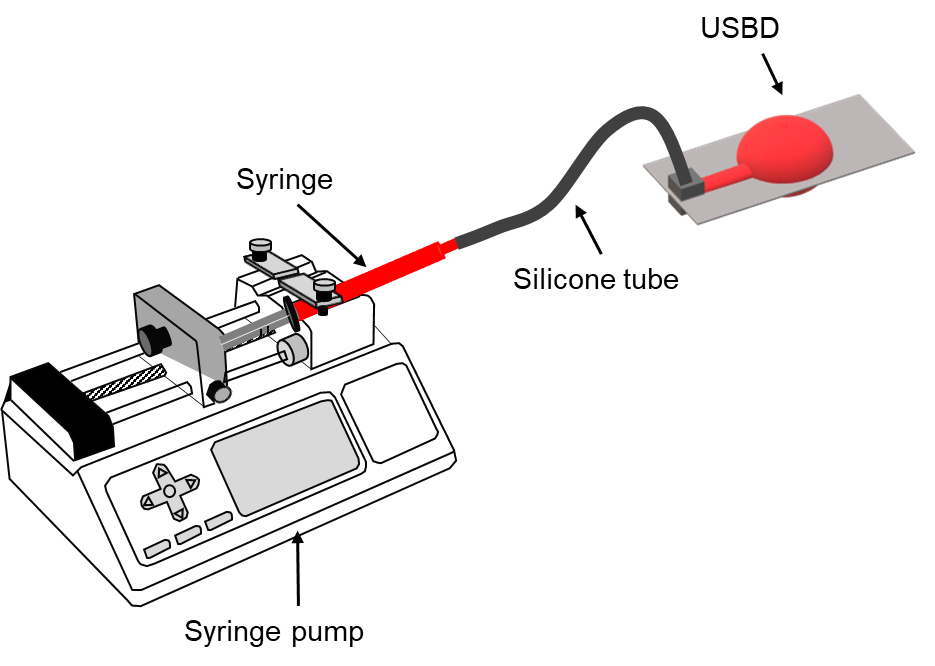


**Table S1.** Loading capacity of the USBDs based on membrane thickness and composition of PDMS. Aliquot sampling was terminated when RB concentration was below the detection limit for each USBD.

| **Device type** | **Membrane thickness (µm) or composition** | **Safe drug loading capacity**  **(µl)** | **Average cumulative percent release (%)** | **Average total time of sustained release (days)** | **Average Release rate**  **(µg/days)** |
| --- | --- | --- | --- | --- | --- |
| **PDMS membrane thickness** | | | | | |
| **USBD255** | 255 | 28 | 58.24 | 147 | 0.446 |
| **USBD178** | 178 | 28 | 69.21 | 113 | 0.676 |
| **USBD140** | 140 | 28 | 80.22 | 113 | 0.990 |
| **USBD115** | 115 | 28 | 82.49 | 113 | 0.996 |
| **USBD96** | 96 | 28 | 90.33 | 79 | 1.115 |
| **USBD81** | 81 | 28 | 95.62 | 68 | 1.536 |
| **USBD73** | 73 | 28 | 96 | 68 | 1.558 |
| **PDMS membrane composition** | | | | | |
| **USBD5** | 1:5 | 28 | 69.16 | 150 | 0.528 |
| **USBD10** | 1:10 | 28 | 69.21 | 116 | 0.676 |
| **USBD15** | 1:15 | 28 | 75 | 116 | 0.759 |
| **USBD20** | 1:20 | 26 | 86.45 | 116 | 0.902 |
| **USBD25** | 1:25 | 26 | 86.149 | 116 | 1.002 |

**Table S2.** Mathematically predicted parameters during zero-order and nearly zero-order release from USBDs based on membrane thickness and composition of PDMS. R_1_^2^ represents coefficient of determination for a zero-order release, and R_2_^2^ represents coefficient of determination for a nearly zero-order release.

| **Device type** | **R_1_^2^** | **Zero-order release (ZOR) period (days)** | **Percent RB release during ZOR period (%)** | **Total drug release during ZOR period (µg)** | **Release rate during ZOR (µg/day)** | **Nearly zero-order release (NZOR) period (days)** | **R_2_^2^** | **Release rate during NZOR (µg/day)** |
| --- | --- | --- | --- | --- | --- | --- | --- | --- |
| **PDMS membrane thickness** | | | | | | | | |
| **USBD255** | **0.996** | **30** | **28.5** | **33.4** | **1.16** | **58** | **0.960** | **0.82** |
| USBD178 | 0.991 | 18 | 32.6 | 38.2 | 1.97 | 38 | 0.962 | 1.39 |
| USBD140 | 0.992 | 18 | 41.84 | 49.0 | 2.03 | 38 | 0.960 | 1.42 |
| USBD115 | 0.991 | 18 | 48.59 | 56.94 | 2.99 | 33 | 0.960 | 2.02 |
| USBD96 | 0.993 | 15 | 52.71 | 61.78 | 3.56 | 24 | 0.960 | 2.75 |
| USBD81 | 0.99 | 15 | 63.04 | 73.88 | 4.21 | 24 | 0.964 | 3.31 |
| USBD73 | 0.99 | 15 | 63.41 | 74.32 | 4.25 | 24 | 0.963 | 3.35 |
| **PDMS membrane composition** | | | | | | | | |
| USBD5 | 0.99 | 12 | 24.57 | 28.79 | 2.085 | 46 | 0.961 | 1.154 |
| USBD10 | 0.991 | 18 | 32.67 | 38.28 | 2.108 | 46 | 0.962 | 1.262 |
| USBD15 | 0.99 | 18 | 35.56 | 41.67 | 2.213 | 46 | 0.961 | 1.434 |
| USBD20 | 0.991 | 21 | 44.78 | 52.48 | 2.241 | 46 | 0.964 | 1.69 |
| USBD25 | 0.992 | 21 | 46.34 | 54.31 | 2.431 | 52 | 0.962 | 1.713 |

**Table S3.** Comparison of previously developed non-biodegradable devices with USBD

| **Authors** | **Application** | **Materials** | **Mechanical properties** | **Cytotoxicity** | **Fibotics capsule thickness (μm)** | **Zero-order release time (days)** | **References** |
| --- | --- | --- | --- | --- | --- | --- | --- |
| Lee et al. | Subcutaneous implant | PMMA | Rigid | No | 903.9 ± 48.91 | 23 | [3] |
| Ji et al. | Subcutaneous implant | PMMA | Rigid | No | 990.9 ± 111.5 | 24 | [4] |
| Nagai et al. | Eye implant | PEGDM/TEGDM | Rigid | Negligible | Nil | No zero- order release | [5] |
| Whyte et al. | Epicardial implant | TPU | Relatively rigid than PDMS | No | ≥ 200 | No zero- order release | [6] |
| Ji et al. | Subcutaneous implant | Molded PDMS | Relatively rigid than membrane-type PDMS | No | 858.818 ± 52.6 | 16 | [9] |
| **Muhammad et al.** | **Subcutaneous implant** | **PDMS (membrane-type)** | **Ultra-soft** | **No** | **53.33 ± 16.02** | **28** | **This study** |

There are implantable drug delivery devices made of different materials for sustained release of drugs. For instance, Lee et al. fabricated an implantable micro-chip for controlled delivery of diclofenac using poly(methyl methacrylate) (PMMA) for 30 days in rats [3]. PMMA has excellent biocompatibility and bio inertness, resulting in no cytotoxicity during implantation. However, due to it rigid mechanical properties, it can induce high inflammation and tissue trauma during and after implantation. As a result, the reported fibrotic capsule was severe with a minimum thickness of 903.9 ± 48.91 µm. Similarly, Ji et al. proposed an implantable drug-delivery chip made of PMMA for prolonged delivery of Diclofenac and Tranilast (anti-fibrotic drug) for 30 days in rats [4]. Due to the intrinsic rigidity of PMMA, the induced fibrotic capsule formation without anti-fibrotic drug was severe with a minimum thickness of 990.9 ± 111.5 µm. After applying Tranilast, the fibrotic capsule thickness was reduced to 273.0 ± 127.2 µm, which was still higher than the average minimum capsule thickness achieved with USBD (53.33 ± 16.02 µm). Nagai et al. developed an implantable device using tri(ethyleneglycol)dimethacrylate (TEGDM) and poly(ethyleneglycol)dimethacrylate (PEGDM) for controlled drug delivery to the posterior segment of the eye. The blend of these polymers results in a rigid implant, which would induce severe fibrotic encapsulation (results were not presented in the manuscript) [5]. Additionally, some monomers of unpolymerized PEGDM and TEGDM and photoinitiator were found to elute from the device and may induce localized and systematic toxicity. Whyte et al. developed a therapeutic epicardial device (Therepi) made of thermoplastic polyurethane (TPU) implanted in rodents for 28 days [6]. TPU is also an FDA approved material for medical implants and it has proven long-term biocompatibility. However, when implanted in rats for 28 days, a severe fibrous capsule thickness was observed with a thickness of more than 200 µm. The main reason of fibrotic encapsulation was that TPU is relatively more rigid with less flexibility and stretchability than PDMS, causing a mismatch between the biological tissues and Therepi. Comparison among previous devices materials with USBD’s material is summarized in Table S3.

In long-term implantable drug delivery devices, there are many factors effecting the device performance including constituent material toxicity, design, shape and size of the device [7,8]. Most the non-biodegradable implants are fabricated from polymers with excellent long-term biocompatibility and with no cytotoxic behaviour as mentioned in the comparison. However, one of the major problems in non-biodegradable long-term implants is fibrotic capsule formation, which greatly affects the device efficacy. All the devices presented here showed no in vivo and in vitro cytotoxicity but the materials’ rigidity, device design, shape and size induced severe fibrotic capsule around the device, which in turn affects the long-term zero-order release. Ji et al. fabricated a soft PDMS-based implantable device to release diclofenac for 30 days in living rats [9]. Although the material used for this device was the same as ours, but due to the moulding techniques, the fabricated device was rigid, subsequently causing severe fibrotic capsule with a thickness of 858.818 ± 52.6 μm (minimum).

**References**

**[1]** A. Raval, J. Parikh, C. Engineer, Mechanism of controlled release kinetics from medical devices, Brazilian J. Chem. Eng. 27 (2010). <https://doi.org/10.1590/S0104-66322010000200001>.

**[2]** J. Shen, D.J. Burgess, In vitro-in vivo correlation for complex non-oral drug products: Where do we stand?, J. Control. Release. 219 (2015). <https://doi.org/10.1016/j.jconrel.2015.09.052>.

**[3]** Lee SH, Park M, Park CG, Kim BH, Lee J, Choi S, et al. Implantable micro-chip for controlled delivery of diclofenac sodium. J Control Release. 2014;196.

**[4]** Ji HB, Hong JY, Kim CR, Min CH, Han JH, Kim MJ, et al. Microchannel-embedded implantable device with fibrosis suppression for prolonged controlled drug delivery. Drug Deliv. 2022;29.

**[5]** Nagai N, Kaji H, Onami H, Ishikawa Y, Nishizawa M, Osumi N, et al. A polymeric device for controlled transscleral multi-drug delivery to the posterior segment of the eye. Acta Biomater. 2014;

**[6]** Whyte W, Roche ET, Varela CE, Mendez K, Islam S, O’Neill H, et al. Sustained release of targeted cardiac therapy with a replenishable implanted epicardial reservoir. Nat Biomed Eng. 2018;2.

**[7]** Veiseh O, Doloff JC, Ma M, Vegas AJ, Tam HH, Bader AR, et al. Size- and shape-dependent foreign body immune response to materials implanted in rodents and non-human primates. Nat Mater. 2015;14.

**[8]** Matlaga BF, Yasenchak LP, Salthouse TN. Tissue response to implanted polymers: The significance of sample shape. J Biomed Mater Res. 1976;10.

**[9]** Ji HB, Kim SN, Lee SH, Huh BK, Shin BH, Lee C, et al. Soft implantable device with drug-diffusion channels for the controlled release of diclofenac. J Control Release. 2020;
